# Synthesis and Utilization of Nitroalkyne Equivalents in Batch and Continuous Flow

**DOI:** 10.1002/anie.201706157

**Published:** 2017-10-04

**Authors:** Peter D. Morse, Timothy F. Jamison

**Affiliations:** ^1^ Department of Chemistry Massachusetts Institute of Technology 77 Massachusetts Ave. Cambridge MA 02139 USA

**Keywords:** cycloaddition, heterocycles, multi-step continuous flow synthesis, nitroalkynes, unstable intermediates

## Abstract

We report a method for overcoming the low stability of nitroalkynes through the development of nitrated vinyl silyltriflate equivalents. Because of their instability, nitroalkynes have only rarely been utilized in synthesis. The reactivity of these silyltriflates, which are prepared in situ, is exemplified by dipolar cycloaddition reactions with nitrones to give highly substituted 4‐nitro‐4‐isoxazolines in high yields. This approach has proven general for several different alkyl and aryl substituted alkynes. In order to minimize the accumulation of potentially hazardous reaction intermediates, we have also developed a continuous flow variant of this method that is capable of carrying out the entire reaction sequence in a good yield and a short residence time.

1‐Nitroalkynes (nitroalkynes) are a family of molecules whose structure and high reactivity give them the potential to serve as versatile synthetic intermediates, especially for the rapid construction of nitrated heterocycles which are prevalent in anti‐microbial agents and next‐generation antibiotics.^1^ Reports on the preparation of nitroalkynes are sparse,^2^ with the first successful synthesis being achieved in 1969 by Viehe and co‐workers, when **1** was prepared by an addition‐elimination sequence (Figure 1).^3^ The nitration of alkynyl stannanes with nitronium tetrafluoroborate (NO_2_BF_4_) and hexafluorophosphate (NO_2_PF_6_) can be used to access alkyl‐substituted nitroalkynes **2**–**4** in moderate yields, though they are noted to rapidly decompose.^2^, ^4^ Physical data has been recorded for 1‐nitro‐2‐phenylacetylene **5**, but a yield for its preparation (4.5 %) has only been recorded once by Kashin et al.^5^ Later, Schmitt and co‐workers showed that bis‐silyl substituted alkynes can also be treated with NO_2_BF_4_ or NO_2_PF_6_ to prepare silyl‐substituted nitroalkynes, which have proven to be uniquely stable members of this class.^6^ In one report, the parent 1‐nitroacetylene was prepared in a 20 % yield using similar conditions and was characterized in situ, as purification was not attempted due to safety considerations.^7^

**Figure 1:**
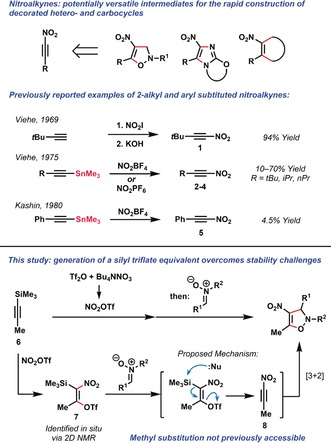
Nitroalkynes as versatile building blocks for organic synthesis, prior methods for nitroalkyne generation, and the strategy employed in this study.

Because of our interest in nitroalkynes as building blocks for constructing biologically active heterocycles, we have set out to develop an improved, high yielding, strategy for accessing members of this class bearing a variety of substituents, both alkyl and aryl, which overcomes previous limitations caused by their instability. Low molecular weight nitroalkynes are presumed to be explosive,^2^ organo‐tin reagents are known to be toxic,^8^ and NO_2_BF_4_ is considered hazardous due to its propensity to release hydrofluoric acid upon contact with water.^9^ These are issues we have also sought to address in this research.

Continuous flow reactors are an attractive alternative to batch reactors in transformations that involve reactive and unstable intermediates. Because only small quantities of starting materials are subjected to reaction conditions at once, safety risks associated with hazardous materials can be minimized.^10^ Therefore, another major goal of this project has been to develop a continuous flow system in which any highly reactive species can be generated and rapidly consumed in‐line. An additional goal has been to utilize safer sources of nitronium ions that can also be generated in‐line, giving in total a three‐step telescoped reaction sequence.

We have found through a series of NMR experiments that nitronium triflate (NO_2_OTf), which is generated in situ from the reaction of triflic anhydride (Tf_2_O) and tetrabutylammonium nitrate (Bu_4_NNO_3_),^11^ adds across 1‐trimethylsilylpropyne **6** to generate silyltriflate **7**, which is stable at or below 0 °C (Figure 2). Key pieces of evidence for this assignment include ^13^C NMR peaks at 149.8 and 149.2 ppm, diagnostic of a nitro‐olefin. We were also able to observe HMBC cross‐peaks between these ^13^C NMR peaks and ^1^H NMR peaks corresponding to a trimethylsilyl group and vinyl methyl group. This species exists as a mixture of *E* and *Z* isomers, which give distinct signals and indicate that the addition is stepwise in nature. Isolation of **7** was not attempted.

**Figure 2:**
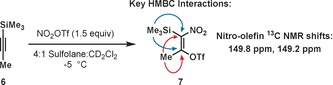
In situ NMR experiments indicate the formation of a vinyl silyltriflate intermediate.

Silyltriflates have served as valuable masked equivalents of other classes of strained or unstable alkynes, such as arynes,^12^ heteroarynes,^13^ cyclic alkynes,^14^ and allenes.^15^ Revealing the appropriate alkyne via elimination in the presence of a reaction partner often gives the desired products in high yields. Having imagined that a similar strategy would be possible using **7**, we were pleased to find that the addition of 3.0 equivalents of nitrone **9** resulted in the rapid formation of the desired 4‐nitro‐4‐isoxazoline **10** in a high yield (Table 1, entry 1). No product formation was observed when 1.0 equivalents of the nitrone were used (entry 2). Allowing the reaction to warm to room temperature resulted in decreased yields, as did the use of 1.0 equivalents of NO_2_OTf (entries 3 and 4). The use of sulfolane as a solvent has also proven critical for obtaining high yields (entry 5). DCM was required as a co‐solvent in order to solubilize the alkyne starting materials. Sulfolane has previously been observed to be uniquely effective for enabling challenging nitration reactions of sensitive substrates.^16^ No product formation was observed when using nitronium trifluoroacetate (NO_2_TFA) (entry 6).

**Table 1:** One‐pot generation of a vinyl silyltriflate and trapping with nitrones via [3+2] cycloaddition. 
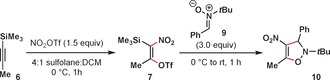

Entry	Conditions	Yield of **10** [%]^[a]^
1	standard conditions	91
2	1.0 equiv of **9** instead of 3.0 equiv **9**	<5
3	0 °C to RT, 1 h instead of 0 °C, 1 h	45
4	1.0 equiv NO_2_OTf instead of 1.5 equiv NO_2_OTf	34
5	1:1 MeNO_2_:DCM instead of 4:1 sulfolane:DCM	37
6	1.5 equiv NO_2_TFA instead of 1.5 equiv NO_2_OTf	<5

[a] Yields were determined by ^1^H NMR analysis using 1,3,5‐trimethoxybenzene as an internal standard.

A notable difference between this methodology and similar transformations involving silyltriflates is that the reaction proceeds in the absence of an activating reagent such as a fluoride source or exogenous base.^18^ Nitrones have been reported to interact appreciably with Lewis acids,^19^ and since multiple equivalents are required we propose that the first is used sacrificially to induce the desired elimination.^20^ This would reveal the putative nitroalkyne intermediate, which we propose rapidly undergoes the observed [3+2] cycloaddition (Figure 1). In one previous case 1‐nitropropyne (**8**) has been directly observed by mass spectroscopy,^6^ but to the best of our knowledge no yield for its direct preparation has been recorded.

With optimized conditions in hand we then explored the scope of nitrone partners (Table 2). A variety of *N‐tert*‐butyl alkynes bearing alkyl, aromatic, and heteroaromatic substituents gave products **10**–**17** in good yields. 5,5‐Dimethyl‐1‐pyrroline *N*‐oxide (DMPO) and an *N*‐benzyl protected nitrone also gave the desired heterocycles **18** and **19**.

**Table 2:** Nitrone scope.^[a]^ 
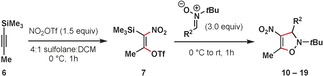

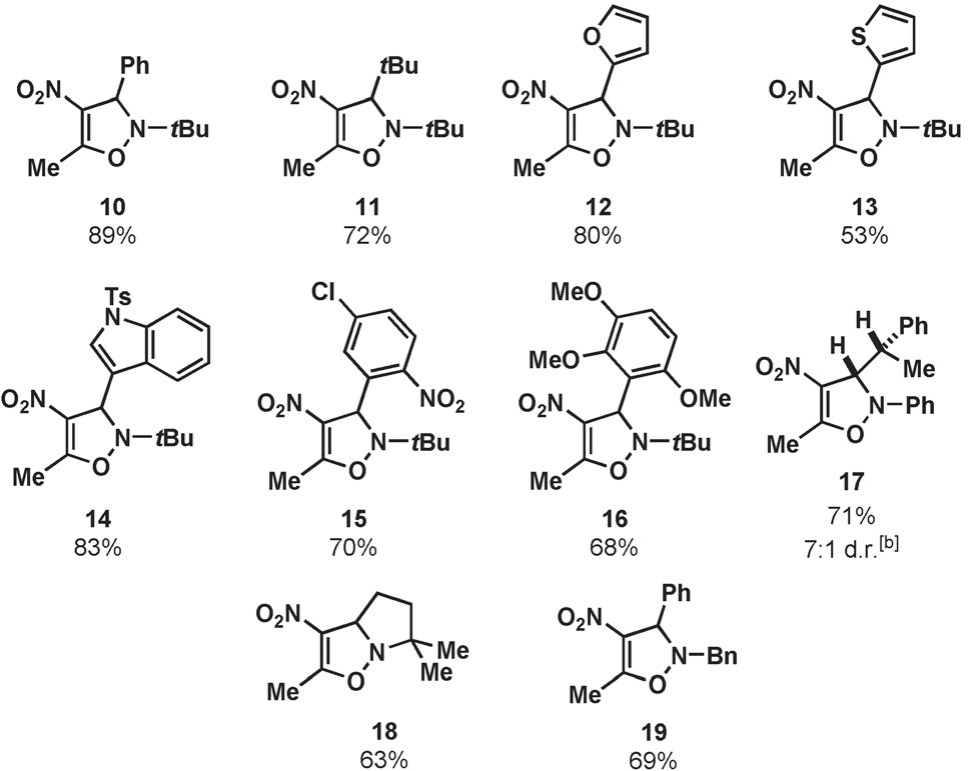

[a] Reactions conducted on 1.0 mmol scale, yields reported are an average of two isolated yields. [b] Diastereoselectivity determined by crude ^1^H NMR, major diastereomer assigned by X‐ray crystallography.^17^

We subsequently investigated the scope of the reaction with regard to the alkyne component (Table 3). Alkynes bearing larger alkyl substituents gave higher yields of the desired products **20**–**22** when reaction times were extended to two hours and allowed to warm to room temperature. Additionally, an X‐ray crystal structure of **22** was obtained which was able to unambiguously confirm the identity and regiochemistry of the product.^21^ Aryl substituted alkynes were viable substrates as well (**23**–**26**).

**Table 3:** Alkyne scope.^[a]^ 
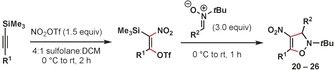

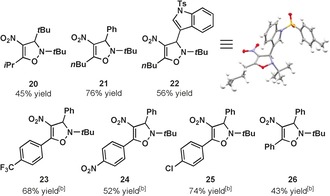

[a] Reactions conducted on 1.0 mmol scale, yields reported are an average of two isolated yields. [b] Step 1 kept at 0 °C, 1 h reaction time.

Many nitroalkynes are presumed to be explosive in nature, as are many sources of nitronium ions.^22^ We therefore designed a continuous flow reactor that would be able to carry out the entire reaction sequence in order to minimize the quantity of hazardous compounds being accumulated (Figure 3). Our initial reactor design consisted of three reservoirs containing stock solutions of NO_2_OTf, alkyne **6**, and nitrone **9**. These were connected to a reactor constructed from two T‐mixers, perfluoralkoxyalkane (PFA) tubing, a back pressure regulator (BPR), and cooling bath. When attempting to minimize the residence time for this sequence, we found that a yield of 60 % of **10** could be obtained with a total residence time of 8.4 minutes. Hypothesizing that this decrease was due to poor or incomplete mixing, we introduced a helical static mixer immediately after the first T‐mixer, which was found to improve the overall yield to 72 % in the same residence time.

**Figure 3:**
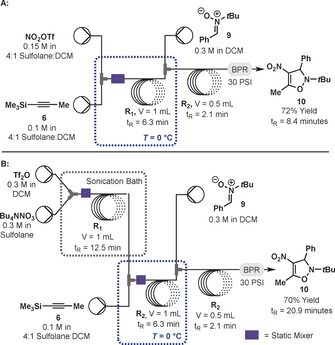
a) Continuous‐flow nitration and [3+2] cycloaddition. b) A continuous‐flow reactor telescoped to include in‐line generation of nitronium triflate.

A second reactor was then designed that allowed for the in‐line preparation of NO_2_OTf. Issues with clogging were initially observed, but quickly solved by moving from a T‐ to a Y‐mixer, though yields were again low. While preparing solutions of NO_2_OTf for batch reactions, we had qualitatively observed that initially Tf_2_O was immiscible with sulfolane, and would remain as a separate phase unless vigorously shaken. We hypothesized that this was the cause of the decreased yields and might also be solved by enhanced mixing. In this case, static mixers were not sufficient. Submerging the reactor in a sonication bath, which we propose aids dissolution by breaking up aggregates of Tf_2_O, improved the yield of **10** to 70 %. This three‐step flow sequence possesses an overall average residence time of only 20.9 minutes, generates both NO_2_OTf and silyltriflate **7** in limited quantities that are used immediately, and provides the desired product in a good overall yield.

In conclusion, we have developed a general method for the preparation and utilization of novel silyltriflates that serve as equivalents of nitroalkynes. The use of this intermediate overcomes the minimal stability of nitroalkynes, and has allowed for the construction of a collection of diverse, functionalized heterocycles in good to high yields. Several alkyne substituents, such as methyl and functionalized phenyl rings, which have not been previously accessible, are now accessible with this methodology. We have addressed safety issues associated with the potentially explosive nature of multiple reaction intermediates by developing flow reactors in which both NO_2_OTf and silyltriflates are generated and consumed in small quantities. Work is currently underway to expand the scope of reaction partners in order to prepare other classes of medicinally relevant heterocycles and carbocycles, as well as gain further insight into the mechanism of the reaction.

## Conflict of interest

The authors declare no conflict of interest.

## Supporting information

As a service to our authors and readers, this journal provides supporting information supplied by the authors. Such materials are peer reviewed and may be re‐organized for online delivery, but are not copy‐edited or typeset. Technical support issues arising from supporting information (other than missing files) should be addressed to the authors.

Supplementary
