# Supplementary material for: Synthesis and Utilization of Nitroalkyne Equivalents in Batch and Continuous Flow
Source: Angew Chem Int Ed Engl. 2017 Oct 4;56(45):13999–4002. doi: 10.1002/anie.201706157 (PMC5698803; doi:10.1002/anie.201706157)
Supplement: Supplementary file 1 — Supplementary [file ANIE-56-13999-s001.pdf]

Angewandte Chemie
Eine Zeitschrift der Gesellschaft Deutscher Chemiker

Supporting Information

Synthesis and Utilization of Nitroalkyne Equivalents in Batch and Continuous Flow

*Peter D. Morse and Timothy F. Jamison**

anie_201706157_sm_miscellaneous_information.pdf

Supporting Information

WILEY-VCH

Table of Contents

Materials and Methods……………………………………………………………………………..…..S-2

Experimental Procedures…………………………………………………………………….………S-3

A. Cycloaddition Experiments……………………………………………………………………S-3
B. Continuous Flow Experiments…………………………………………………………...…S-9
C. NMR Experiments……………………………………………………………………….....S-13

References……………………………………………………………………………………….…S-14

NMR Spectra……………………………………………………………………………………….S-15

WILEY-VCH

Materials and Methods

All reactions were performed under an inert atmosphere of nitrogen with the exlusion of moisture from reagents and glassware unless otherwise noted. All cycloaddition reactions were set up in a glovebox (MBraun Unilab) filled with dry nitrogen. Tf_2O (>99% purity, ampule) was purchased from Sigma-Aldrich and stored in the glovebox at -20 °C. Tetrabutylammonium nitrate was purchased from Sigma-Aldrich and stored in the glovebox after drying overnight in a vacuum desiccator over $CaSO_4$. All other commercially available reagents were used as received. Commercially available chemicals were purchased from either Sigma-Aldrich Chemical Company (Milwaukee, WI), Alfa Aesar (Ward Hill, MA), Acros Organics (Pittsburgh, PA), or TCI America (Portland, OR). Sulfolane was stirred over 4Å molecular sieves, distilled, and stored in the glovebox. 3% v/v DCM was added to the dried sulfolane in order to ensure that it was a liquid at ambient temperature. All other solvents were dried by passing through a column of activated alumina on an SG Water solven purification system. Analytical thin-layer chromatography (TLC) was performed on 0.2 mm coated Science silica gel (EM 60-F254) plates. Visualization was accomplished with UV light (254 nm) and exposure to para-anisaldehyde or $KMnO_4$ solution followed by heating. Column chromatography was carried out on a Biotage Isolera flash chromatography system using SNAP KP-Sil columns (silica gel, average particle size 50 µm).

For flow experiments, backpressure regulators (BPRs) were purchased from Zaiput Flow Technologies. The reactors were constructed from high-purity perfluoroalkoxy (PFA) tubing with 1/16” OD and 0.03″ ID and PEEK superflangeless fittings purchased from IDEX Health & Science Technologies. Harvard Apparatus PhD Ultra syringe pumps were used to pump reagents and solutions from 8-mL high-pressure stainless steel syringes with 1/16” SWAGELOCK® from Harvard Apparatus. Helical PTFE static mixers purchased from Stamixco were used for enhanced mixing. Cooling was accomplished by submerging tubing in an ice/water bath. Sonication was accomplished by submerging tubing in a VWR Model 150D Sonicator.

^1H and ^{13}C NMR spectra were recorded in $CDCl_3$ solutions or mixtures of sulfolane and CD_2CL_2 using a Bruker Avance (400 MHz for ^1H, 100 MHz for ^{13}C), or JEOL 500 MHz (500MHz for ^1H, 126MHz ^{13}C) Chemical shifts (^1H and ^{13}C) are reported in parts per million and referenced to the residual solvent peak (for $CDCl_3$, δ = 7.27 ppm, 77.0 ppm respectively). The following designations are used to describe multiplicities: s (singlet), d (doublet), t (triplet), dd (doublet of doublets), ddd (doublet of doublet of doublets), ddt (doublet of doublet of triplets), td (triplet of doublets). 1,3,5-Trimethoxybenzene (>97%, Sigma-Aldrich) was used as an internal standard for quantification. IR spectra were obtained on an Agilent Cary 630 FT-IR spectrometer equipped with an ATR accessory. High-resolution mass spectrometry data were acquired by the Department of Chemistry Instrumentation Facility, Massachusetts Institute of Technology on a Bruker Daltonics APEXIV 4.7 Tesla FT-ICR Mass Spectrometer.

WILEY-VCH

Experimental Procedures

A. Nitroalkyne Trapping Experiments

Representative Procedure (Preparation of isoxazoline 16 is used as an example).

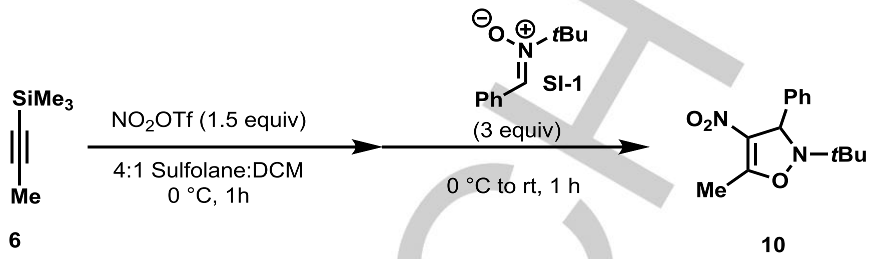

Isoxazolidine (10). To a stirred solution of tetrabutylammonium nitrate (456.7 mg, 1.50 mmol, 1.5 equiv) in 4:1 Sulfolane:DCM was added triflic anhydride (252 µL, 1.50 mmol, 1.5 equiv). Vigorous stirring is initially required to dissolve the triflic anhydride, and a pale yellow color is initially observed that quickly fades. The reaction vessel was sealed, removed from the glove box, and cooled for 15 minutes in an ice water bath. 1-(trimethylsilyl)propyne 9 (148 µL, 1.00 mmol, 1.0 equiv) was added to the reaction dropwise via syringe. In a separate, flame-dried vessel, nitrone SI-1 (531.8 mg, 3.0 mmol, 3.0 equiv) was dissolved in 5 mL DCM. After the reaction was stirred for 1 h, the solution of nitrone was added dropwise to the reaction, which was then allowed to warm to room temperature and stir for an additional hour. The reaction mixture was then concentrated under reduced pressure to remove DCM. The remaining sulfolane solution was then diluted with water and extracted 4 times with methyl tert-butyl ether (MTBE). The combined organic layers were then washed 4 times with ca. 5 mL water, dried with magnesium sulfate, and concentrated under reduced pressure The crude reaction mixture was purified by flash chromatography (Biotage KPs-sil 25g 10-40% EtOAc in hexanes) to afford 16 as a solid (89% yield, average of two experiments).
Rf 0.56 (1:3 EtOAc:hexanes)
1H NMR (500 MHz, Chloroform-d) δ 7.42 (d, J = 7.3 Hz, 2H), 7.34 (t, J = 7.4 Hz, 2H), 7.27 (dd, J = 15.1, 7.8 Hz, 1H), 5.56 (s, 1H), 2.50 (s, 3H), 1.16 (s, 9H).
13C NMR (126 MHz, Chloroform-d) δ 164.65, 140.45, 128.60, 128.58, 128.27, 128.00, 65.86, 62.10, 24.76, 13.47.
IR (film): 2981, 2938, 2717, 2333, 1631, 2458, 1349, 1308, 1257, 1230
HRMS (m/z) [M + H]+ calculated for C14H18N2O3, 263.1390; found: 263.1383

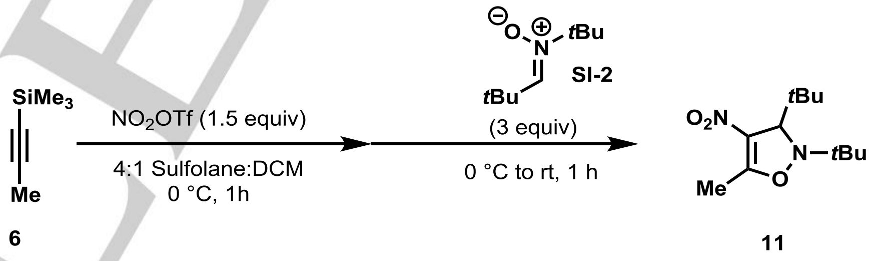

Isoxazolidine (11). Purification by flash chromatography (Biotage KPs-sil 25g 10-40% EtOAc in hexanes) afforded **11** as a clear crystalline solid (72% yield, average of two experiments).
R_f 0.68 (1:3 EtOAc:hexanes)
^{1}H NMR (400 MHz, Chloroform-d) δ 4.29 (s, 1H), 2.45 (s, 3H), 1.10 (s, 9H), 0.91 (s, 9H).
^{13}C NMR (126 MHz, Chloroform-d) δ 167.07, 126.70, 76.75, 68.37, 61.46, 37.51, 25.88, 24.98, 13.12.
IR (film): 2962, 2871, 2359, 2341, 1643, 1475, 1356, 1259, 1232.
HRMS (*m/z*) $[M + H]^+$ calculated for $C_{12}H_{22}N_2O_3$: 243.1703; found: 243.1698

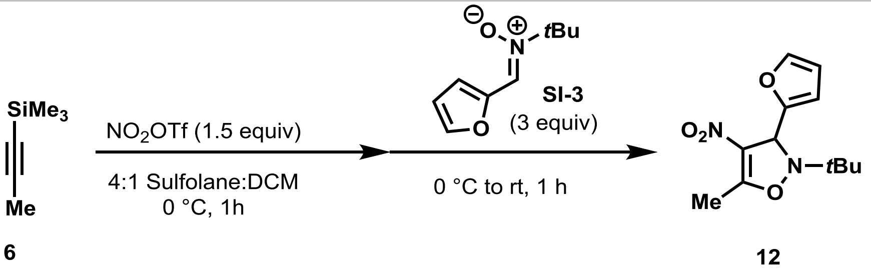

Isoxazolidine (12). Purification by flash chromatography (Biotage KPs-sil 25g 10-40% EtOAc in hexanes) afforded **12** as a clear solid (80% yield, average of two experiments).
R_f 0.53 (1:3 EtOAc:hexanes)
1H NMR (500 MHz, Chloroform-d) δ 7.40 (s, 1H), 6.34 (d, J = 17.6 Hz, 2H), 5.72 (s, 1H), 2.53 (s, 3H), 1.21 (d, J = 1.8 Hz, 9H).
^{13}C NMR (126 MHz, Chloroform-d) δ 165.89, 151.74, 142.85, 124.63, 110.61, 108.08, 62.09, 59.39, 24.50, 13.43, 13.43.
IR (film): 3120, 2980, 2939, 2361, 1639, 1475, 1361, 1306, 1257, 1239
HRMS (*m/z*) $[M + H]^+$ calculated for $C_{12}H_{16}N_2O_4$: 253.1183; found: 253.1172

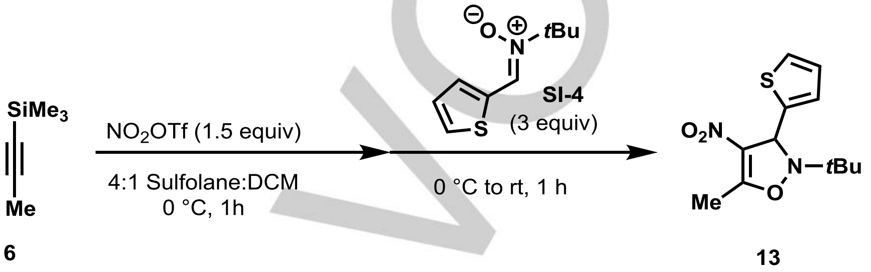

Isoxazolidine (13). Purification by flash chromatography (Biotage KPs-sil 25g 10-40% EtOAc in hexanes) afforded **13** as a yellow solid (53% yield, average of two experiments).
R_f 0.50 (1:3 EtOAc:hexanes)
^{1}H NMR (500 MHz, Chloroform-d) δ 7.34 (s, 1H), 7.27 (s, 2H), 7.17 (s, 1H), 5.74 (d, J = 2.8 Hz, 1H), 2.49 (s, 3H), 1.18 (s, 9H).
^{13}C NMR (126 MHz, Chloroform-d) δ 164.99, 141.55, 126.93, 126.17, 125.57, 123.35, 77.41, 77.16, 76.91, 62.05, 61.27, 24.77, 13.55.
IR (film): 3103, 2975, 2937, 2360, 1638, 1473, 1344, 1309, 1257, 1235, 1202
HRMS (*m/z*) $[M + H]^+$ calculated for $C_{12}H_{16}N_2O_3S$: 269.0954; found: 269.0943

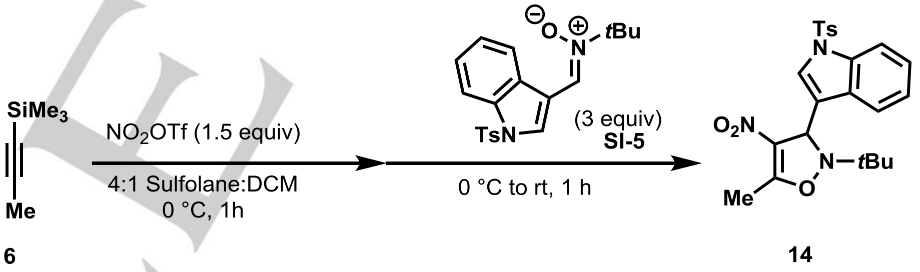

Isoxazolidine (14). Purification by flash chromatography (Biotage KPs-sil 25g 10-50% EtOAc in hexanes) afforded **14** as a solid (83% yield, average of two experiments).
R_f 0.36 (1:3 EtOAc:hexanes)
1H NMR (500 MHz, Chloroform-d) δ 7.93 (d, J = 8.3 Hz, 1H), 7.72 (d, J = 8.4 Hz, 3H), 7.70 (s, 1H), 7.62 (d, J = 7.9 Hz, 1H), 7.30 (t, J = 7.7 Hz, 1H), 7.22 (t, J = 7.5 Hz, 4H), 5.88 (s, 1H), 2.52 (s, 3H), 2.33 (s, 3H), 1.18 (s, 9H).
^{13}C NMR (126 MHz, Chloroform-d) δ 164.82, 144.99, 135.51, 134.83, 129.88, 128.71, 126.78, 126.17, 125.61, 124.74, 123.31, 121.00, 120.48, 113.77, 77.25, 77.00, 76.75, 62.10, 59.30, 24.54, 21.55, 13.40.
IR (film): 3053, 2977, 2937, 2360, 1734, 1641, 1597, 1565, 1475, 1446, 1359, 1259
HRMS (*m/z*) $[M + H]^+$ calculated for $C_{23}H_{25}N_3O_5S$: 456.1588; found: 456.1579

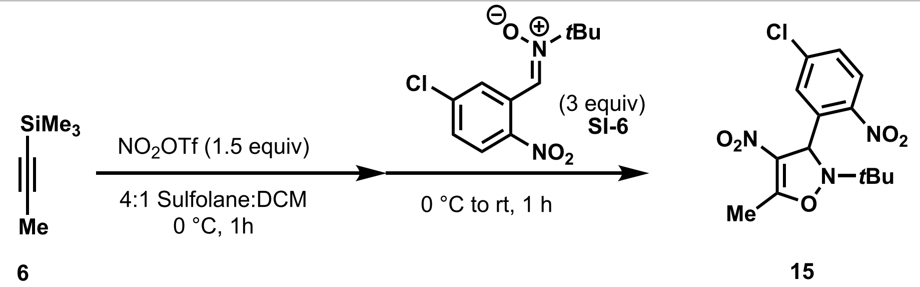

Isoxazolidine (15). Purification by flash chromatography (Biotage KPs-sil 25g 10-40% DCM in hexanes) afforded **16** as a white solid (70% yield, average of two experiments).
R_f 0.40 (1:1 DCM:hexanes)
^{1}H NMR (500 MHz, Chloroform-d) δ 8.25 – 7.63 (m, 2H), 7.40 (dd, J = 8.7, 2.4 Hz, 1H), 6.72 (s, 1H), 2.50 (s, 3H), 1.22 (s, 9H).
^{13}C NMR (126 MHz, Chloroform-d) δ 165.07, 147.59, 139.57, 137.24, 131.22, 129.17, 128.17, 125.84, 62.70, 60.04, 24.68, 13.54.
IR (film): 2977, 2905, 1642, 1478, 1445, 1359, 1310, 1238, 1206
HRMS (*m/z*) [M + H]$^+$ calculated for $C_{14}H_{16}ClN_3O_5$: 342.0857; found: 342.0804

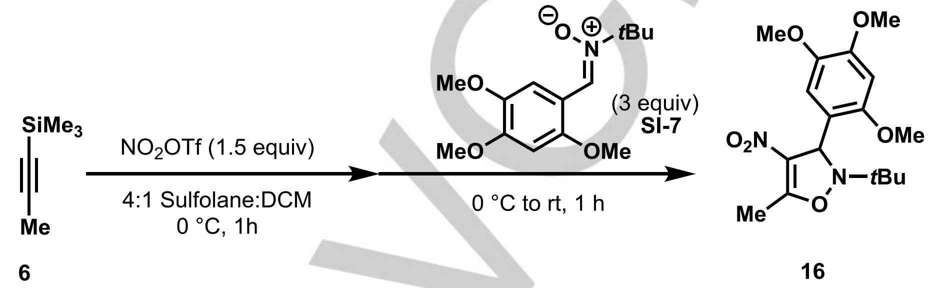

Isoxazolidine (16). Purification by flash chromatography (Biotage KPs-sil 25g 25-75% EtOAc in hexanes) afforded **17** as a white solid (68% yield, average of two experiments).
R_f 0.27 (1:1 EtOAc:hexanes)
^{1}H NMR (500 MHz, Chloroform-d) δ 6.81 (s, 1H), 6.51 (s, 1H), 6.05 (s, 1H), 3.89 (s, 6H), 3.83 (s, 3H), 2.53 (s, 3H), 1.17 (s, 9H).
^{13}C NMR (126 MHz, Chloroform-d) δ 164.87, 151.65, 149.88, 143.76, 128.14, 120.24, 112.43, 97.98, 62.12, 59.18, 57.24, 56.79, 56.14, 24.67, 13.63.
IR (film): 2923, 2865, 1594, 1490, 1449, 1399, 1326
HRMS (*m/z*) [M + H]$^+$ calculated for $C_{17}H_{24}N_2O_6$: 353.1707; found: 353.1720

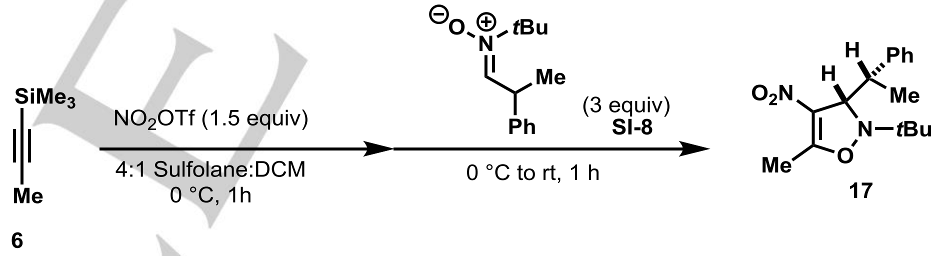

Isoxazolidine (17). Purification by flash chromatography (Biotage KPs-sil 25g 25-75% EtOAc in hexanes) afforded **17** as a white solid (71% yield, average of two experiments).
R_f 0.41 (1:3 EtOAc:hexanes)
^{1}H NMR (500 MHz, Chloroform-d) δ 7.21 (m, 5H), 4.67 (d, *J* = 3.3 Hz, 1H), 3.39 (qd, *J* = 7.2, 3.3 Hz, 1H), 2.00 (s, 3H), 1.37 (d, *J* = 7.2 Hz, 3H), 1.11 (s, 9H).
^{13}C NMR (126 MHz, Chloroform-d) δ 166.91, 141.39, 128.41, 127.55, 126.62, 65.55, 61.24, 40.80, 24.97, 16.94, 12.48.
IR (film): 3984, 2879, 1633, 1459, 1356, 1287, 1257, 1236
MS (*m/z*) [M + H]$^+$ calculated for $C_{17}H_{24}N_2O_6$: 291.37; found: 291.41

WILEY-VCH

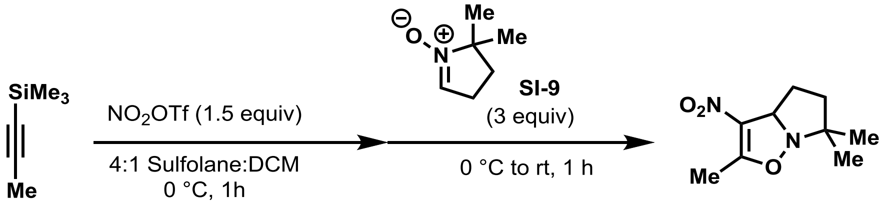

6 18

Isoxazolidine (18). Purification by flash chromatography (Biotage KPs-sil 25g 10-50% EtOAc in hexanes) afforded **15** as a clear oil (63% yield, average of two experiments).

R_f 0.42 (1:3 EtOAc:hexanes)

1H NMR (500 MHz, Chloroform-d) δ 5.06 (dd, J = 8.0, 2.8 Hz, 1H), 2.40 (s, 3H), 2.26 (ddt, J = 13.5, 11.6, 7.8 Hz, 2H), 2.05 (ddt, J = 13.4, 8.4, 2.4 Hz, 1H), 1.78 (ddd, J = 12.7, 7.6, 2.0 Hz, 1H), 1.68 (td, J = 12.2, 8.4 Hz, 1H), 1.40 (s, 3H), 1.13 (s, 3H).

^{13}C NMR (126 MHz, Chloroform-d) δ 164.65, 128.18, 70.80, 67.71, 34.56, 31.50, 25.64, 23.06, 13.31.

IR (film): 3053, 2977, 2937, 2360, 1734, 1641, 1597, 1565, 1475, 1446, 1359, 1259

HRMS (*m/z*) $[M + H]^+$ calculated for $C_9H_{14}N_2O_3$: 199.1077; found: 199.1073

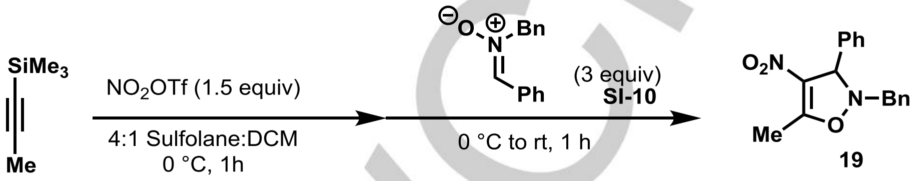

6 19

Isoxazolidine (19). Purification by flash chromatography (Biotage KPs-sil 25g 10-50% EtOAc in hexanes) afforded **15** as a white solid (69% yield, average of two experiments).

R_f 0.54 (1:3 EtOAc:hexanes)

1H NMR (500 MHz, Chloroform-d) δ 7.28 (m, 10H), 5.38 (s, 1H), 4.26 (dd, J = 97.3, 13.1 Hz, 2H), 2.46 (s, 3H).

^{13}C NMR (126 MHz, Chloroform-d) δ 164.65, 128.18, 70.80, 67.71, 34.56, 31.50, 25.64, 23.06, 13.31.

IR (film): 3062, 3031, 2360, 1734, 1700, 1636, 1556, 1475, 1455, 1356, 1242

MS (*m/z*) $[M + H]^+$ calculated for $C_9H_{14}N_2O_3$: 297.12; found: 297.20

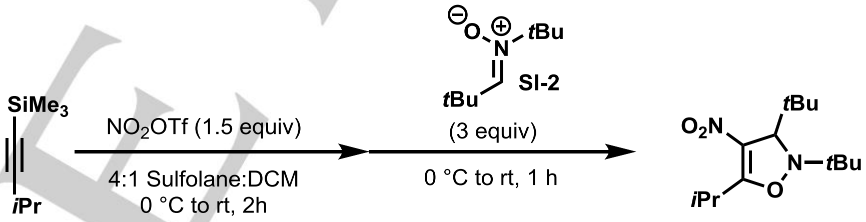

SI-11 20

Isoxazolidine (18). Purification by flash chromatography (Biotage KPs-sil 25g 5-40% EtOAc in hexanes) afforded **18** as a clear oil (45% yield, average of two experiments).

R_f 0.67 (1:3 EtOAc:hexanes)

^{1}H NMR (500 MHz, Chloroform-d) δ 4.30 (s, 1H), 3.81 (p, *J* = 7.0 Hz, 1H), 1.28 (dd, *J* = 21.8, 7.0 Hz, 7H), 1.11 (s, 6H), 0.90 (s, 6H).

^{13}C NMR (126 MHz, Chloroform-d) δ 173.85, 77.25, 77.00, 76.75, 68.50, 61.51, 37.51, 27.61, 25.87, 24.99, 20.18, 18.30.

IR (film): 2974, 2930, 1491, 1481, 1323, 1303, 1251, 1118

HRMS (*m/z*) $[M + H]^+$ calculated for $C_{14}H_{27}N_2O_3$: 271.2022; found: 271.2013

WILEY-VCH

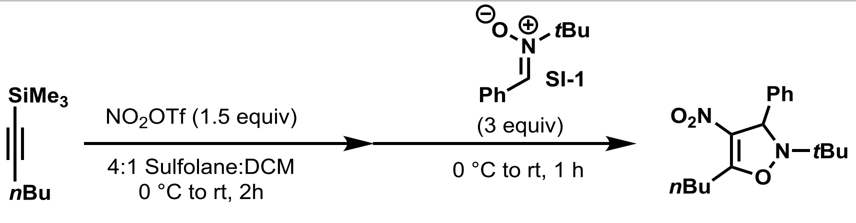

21

Isoxazolidine (19). Purification by flash chromatography (Biotage KPs-sil 25g 3-25% EtOAc in hexanes) afforded **19** as a clear oil (76% yield, average of two experiments).

R_f 0.52 (1:9 EtOAc:hexanes)

1H NMR (500 MHz, Chloroform-d) δ 7.42 (d, J = 7.2 Hz, 2H), 7.35 (t, J = 7.5 Hz, 2H), 7.32 – 7.27 (m, 1H), 5.58 (s, 1H), 2.94 (ddd, J = 8.1, 6.5, 2.7 Hz, 2H), 1.73 (qd, J = 7.6, 2.5 Hz, 2H), 1.55 – 1.42 (m, 2H), 1.18 (s, 9H), 1.00 (t, J = 7.4 Hz, 3H).

^{13}C NMR (126 MHz, Chloroform-d) δ 168.15, 140.54, 128.46, 128.09, 127.86, 65.85, 61.98, 28.31, 26.91, 24.68, 22.59, 13.63.

IR (film): 2963, 2935, 2874, 1630, 1478, 1427, 1359, 1259, 1238, 1203

HRMS (*m/z*) $[M + H]^+$ calculated for $C_{17}H_{24}N_2O_3$: 305.1860; found: 305.1857

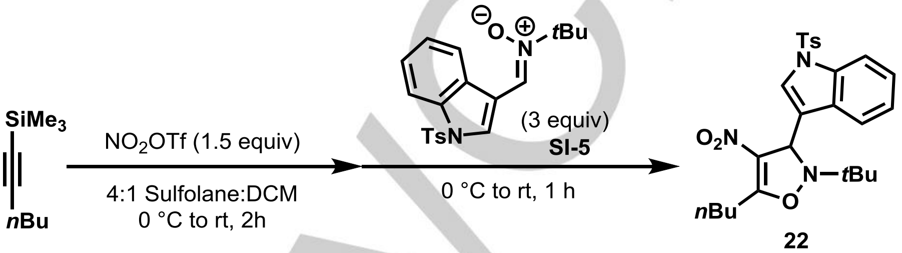

22

Isoxazolidine (20). Purification by flash chromatography (Biotage KPs-sil 25g 6-50% EtOAc in hexanes) afforded **20** as a crystalline solid (83% yield, average of two experiments). X-ray quality crystals were grown from the slow evaporation of a mixture of toluene and DCM over 24 h.

R_f 0.67 (1:3 EtOAc:hexanes)

^{1}H NMR (500 MHz, Chloroform-d) δ 4.30 (s, 1H), 3.81 (p, J = 7.0 Hz, 1H), 1.28 (dd, J = 21.8, 7.0 Hz, 7H), 1.11 (s, 6H), 0.90 (s, 6H).

^{13}C NMR (126 MHz, Chloroform-d) δ 173.85, 68.50, 61.51, 37.51, 27.61, 25.87, 24.99, 20.18, 18.30.

IR (film): 2975, 2874, 2363, 1633, 1478, 1367, 1250, 1200.

HRMS (*m/z*) $[M + H]^+$ calculated for $C_{26}H_{31}N_3O_5S$: 498.2057; found: 498.2091

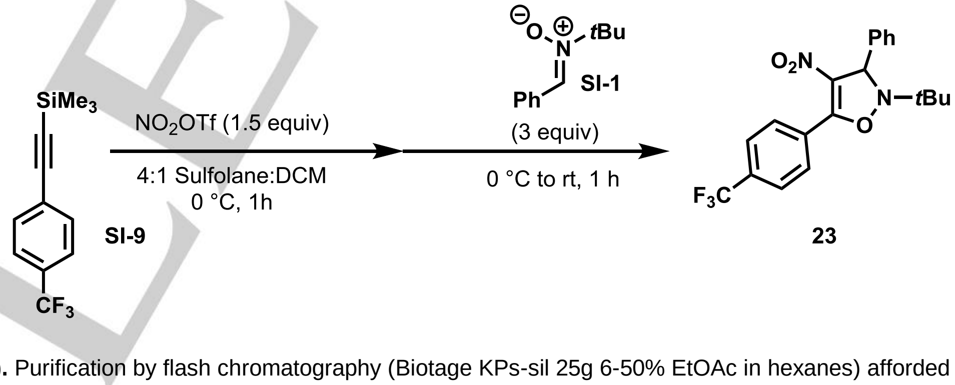

23

Isoxazolidine (21). Purification by flash chromatography (Biotage KPs-sil 25g 6-50% EtOAc in hexanes) afforded **21** as a yellow foam (68% yield, average of two experiments).

R_f 0.58 (1:3 EtOAc:hexanes)

^{1}H NMR (500 MHz, Chloroform-d) δ 7.98 (d, J = 8.1 Hz, 2H), 7.78 (d, J = 8.3 Hz, 2H), 7.53 (d, J = 7.4 Hz, 2H), 7.40 (t, J = 7.5 Hz, 2H), 7.35 (t, J = 7.3 Hz, 1H), 5.82 (s, 1H), 1.28 (s, 9H).

^{13}C NMR (126 MHz, Chloroform-d) δ 159.35, 140.13, 134.04, 133.78, 130.32, 130.31, 128.70, 128.68, 128.46, 127.84, 125.38, 125.35, 67.01, 62.50, 24.76.

IR (film): 2979, 1636, 1612, 1486, 1411, 1351, 1319, 1257.

HRMS (*m/z*) $[M + H]^+$ calculated for $C_{20}H_{19}F_3N_2O_3$: 393.1421; found: 393.1423

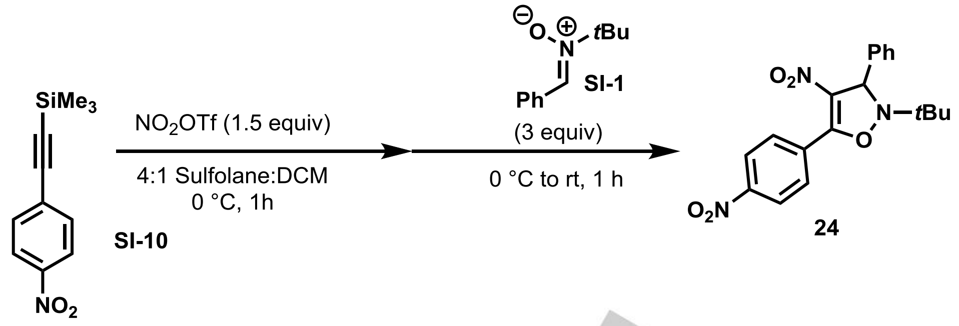

Isoxazolidine (22). Purification by flash chromatography (Biotage KPs-sil 25g 6-50% EtOAc in hexanes) afforded **22** as a yellow foam (52% yield, average of two experiments).

R_f 0.48 (1:3 EtOAc:hexanes)

1H NMR (500 MHz, Chloroform-d) δ 8.37 (d, J = 8.8 Hz, 2H), 8.03 (d, J = 8.9 Hz, 2H), 7.53 (d, J = 7.1 Hz, 2H), 7.41 (t, J = 7.5 Hz, 2H), 7.36 (d, J = 7.2 Hz, 1H), 5.83 (s, 1H), 1.28 (s, 9H).

^{13}C NMR (126 MHz, Chloroform-d) δ 158.21, 149.71, 139.89, 131.18, 131.06, 128.75, 128.58, 128.33, 127.87, 123.48, 67.07, 62.61, 24.77.

IR (film): 2980, 2365, 1637, 1594, 1527, 1491, 1348, 1258.

HRMS (*m/z*) $[M + H]^+$ calculated for $C_{19}H_{19}N_3O_5$: 370.1397; found: 370.1399

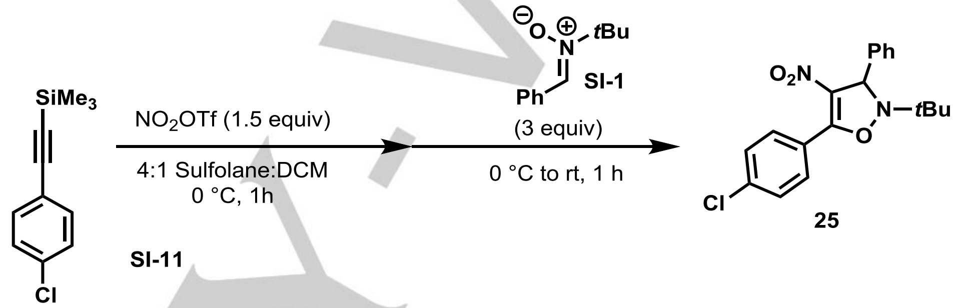

Isoxazolidine (23). Purification by flash chromatography (Biotage KPs-sil 25g 6-50% EtOAc in hexanes) afforded **23** as a yellow foam (74% yield, average of two experiments).

R_f 0.56 (1:3 EtOAc:hexanes)

^{1}H NMR (500 MHz, Chloroform-d) δ 7.85 (d, J = 8.2 Hz, 2H), 7.51 (t, J = 8.3 Hz, 4H), 7.39 (t, J = 7.5 Hz, 2H), 7.34 (t, J = 7.3 Hz, 1H), 5.79 (s, 3H), 1.27 (s, 9H).

^{13}C NMR (126 MHz, Chloroform-d) δ 160.16, 140.50, 139.14, 131.51, 128.92, 128.82, 128.51, 127.96, 127.22, 123.61, 67.13, 62.56, 24.93.

IR (film): 3063, 3033, 3976, 2936, 1618, 1592, 1486, 1402, 1344, 1255, 1205

HRMS (*m/z*) $[M + H]^+$ calculated for $C_{19}H_{19}ClN_2O_3$: 359.1157; found: 359.1151

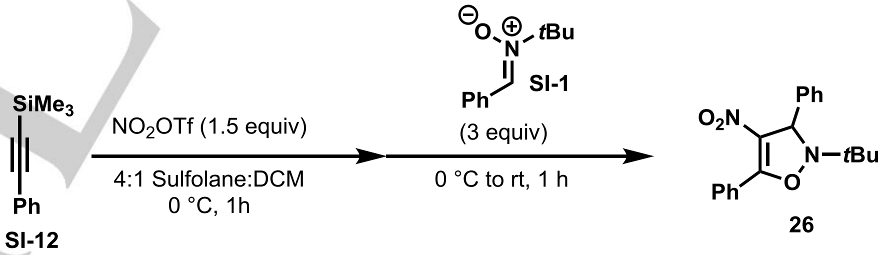

Isoxazolidine (24). Purification by flash chromatography (Biotage KPs-sil 25g 6-50% EtOAc in hexanes) afforded **24** as a yellow foam (43% yield, average of two experiments).

R_f 0.54 (1:3 EtOAc:hexanes)

^{1}H NMR (500 MHz, Chloroform-d) δ 7.90 (d, J = 8.0 Hz, 2H), 7.61 (t, J = 7.5 Hz, 1H), 7.54 (t, J = 7.8 Hz, 3H), 7.40 (t, J = 7.5 Hz, 2H), 7.34 (t, J = 7.3 Hz, 1H), 5.81 (s, 1H), 1.29 (s, 9H).

^{13}C NMR (126 MHz, Chloroform-d) δ 161.52, 140.71, 132.77, 130.11, 128.79, 128.51, 128.41, 127.97, 127.96, 125.26, 67.10, 62.50, 24.93.
IR (film): 2975, 2840, 2363, 1701, 1594, 1544, 1477, 1456, 1347, 1203
HRMS (*m/z*) $[M + H]^+$ calculated for $C_{14}H_{18}N_2O_3$: 325.1547; found: 325.1546

Preparation of Nitrones

Nitrones **SI-1** – **SI-8** and **SI-10** were prepared according to the procedure reported by Garg et al.[1] Purification and characterization of new compounds **SI-6** and **SI-7** are given below:

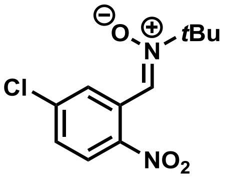

SI-6

Nitrone (SI-6). Purification by flash chromatography (Biotage KPs-sil 25g 6-50% EtOAc in hexanes) afforded **SI-7** as a yellow solid (64% yield).
R_f 0.38 (1:3 EtOAc:hexanes)
^1H NMR (500 MHz, Chloroform-d) δ 9.20 (s, 1H), 7.96 (s, 1H), 6.49 (s, 1H), 3.93 (s, 6H), 3.86 (s, 3H), 1.60 (s, 9H).
^{13}C NMR (126 MHz, Chloroform-d) δ 140.35, 129.70, 129.22, 126.67, 126.37, 126.37, 123.28, 73.23, 28.31.
IR (film): 3103, 2978, 2261, 1596, 1560, 1512, 1455, 1361, 1317
HRMS (*m/z*) $[M + H]^+$ calculated for $C_{11}H_{13}ClN_2O_3$: 256.0615; found: 256.0609

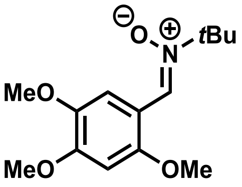

SI-7

Nitrone (SI-7). Purification by flash chromatography (Biotage KPs-sil 25g 25-75% EtOAc in hexanes) afforded **SI-7** as a white solid (70% yield).
R_f 0.09 (1:3 EtOAc:hexanes)
^1H NMR (500 MHz, Chloroform-d) δ 9.27 (d, J = 2.3 Hz, 1H), 8.27 (s, 1H), 8.01 (d, J = 8.8 Hz, 1H), 7.46 (dd, J = 8.8, 2.4 Hz, 1H), 1.63 (s, 9H).
^{13}C NMR (126 MHz, Chloroform-d) δ 153.06, 150.99, 142.29, 124.79, 112.39, 111.42, 95.80, , 70.46, 56.50, 56.24, 56.06, 28.51.
IR (film): 3106, 3040, 2969, 1603, 1569, 1496, 1466, 1291
HRMS (*m/z*) $[M + H]^+$ calculated for $C_{14}H_{21}NO_4$: 267.1471; found: 267.1474

NMR Experiments

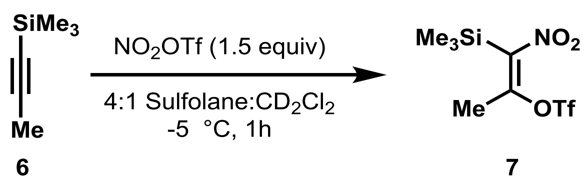

Preparation of 7: 304.5 mg Bu_4NNO_3 (1 mmol) was dissolved in 3 mL sulfolane and 1mL CD_2Cl_2 a dry 5 mL volumetric flask in a nitrogen-filled glovebox. 168 µL Tf_2O was added dropwise and additional sulfolane was added to reach a final volume of 5 mL, giving a .2 M solution of NO_2OTf (1.5 equiv). A 1 mL aliquot was transferred to a dry NMR tube, which was then capped, taped shut, and removed from the glovebox. The sample was chilled in a salt/ice bath for 10 minutes, then 19.7 µL **6** (0.133 mmol, 1 equiv) was added by injecting through the cap, which was quickly taped and the sample transferred to a Varian Inova-500 NMR spectrometer cooled to -5 °C. ^{1}H, ^{13}C, HSQC, HMBC, and COSY spectra were acquired.

7 (*Z*)
^{1}H NMR (500 MHz, Methylene Chloride-d2) δ 2.21 (s, 3H), 0.15 (s, 9H).
^{13}C NMR (126 MHz, Methylene Chloride-d2) δ 149.23, 148.51, 18.03, -2.79.

7 (*E*)
^{1}H NMR (500 MHz, Methylene Chloride-d2) δ 2.05 (s, 3H), 0.13 (s, 9H).
^{13}C NMR (126 MHz, Methylene Chloride-d2) δ 149.07, 148.40, 16.89, -2.82.

WILEY-VCH

Figure S1. ^{1}H NMR and ^{13}C spectra of **7**.

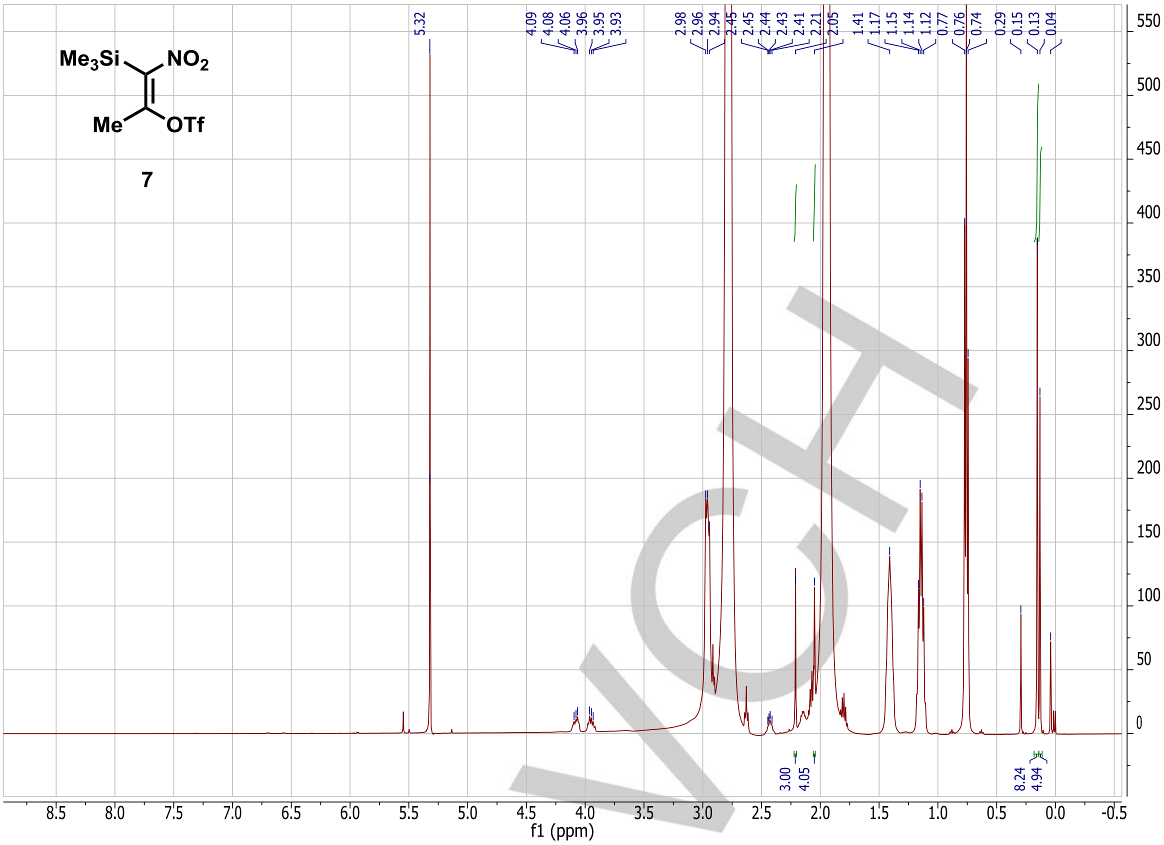

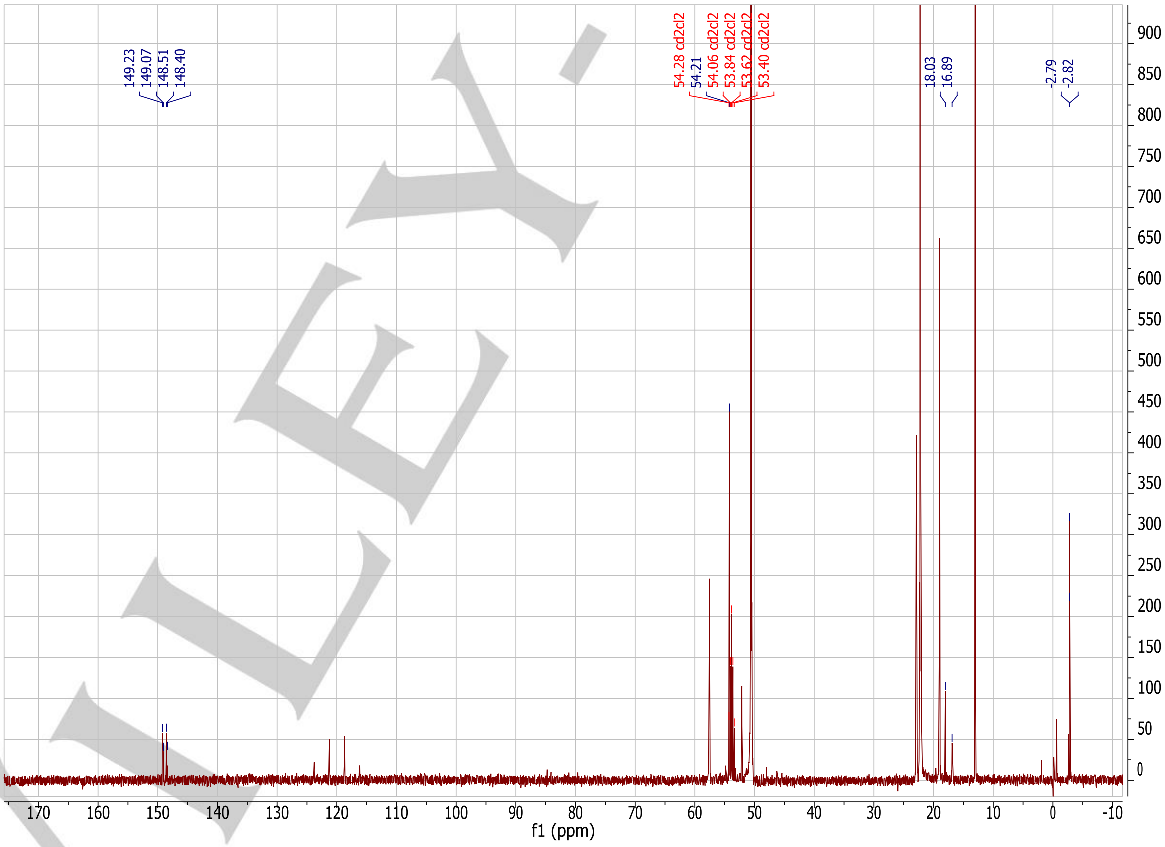

WILEY-VCH

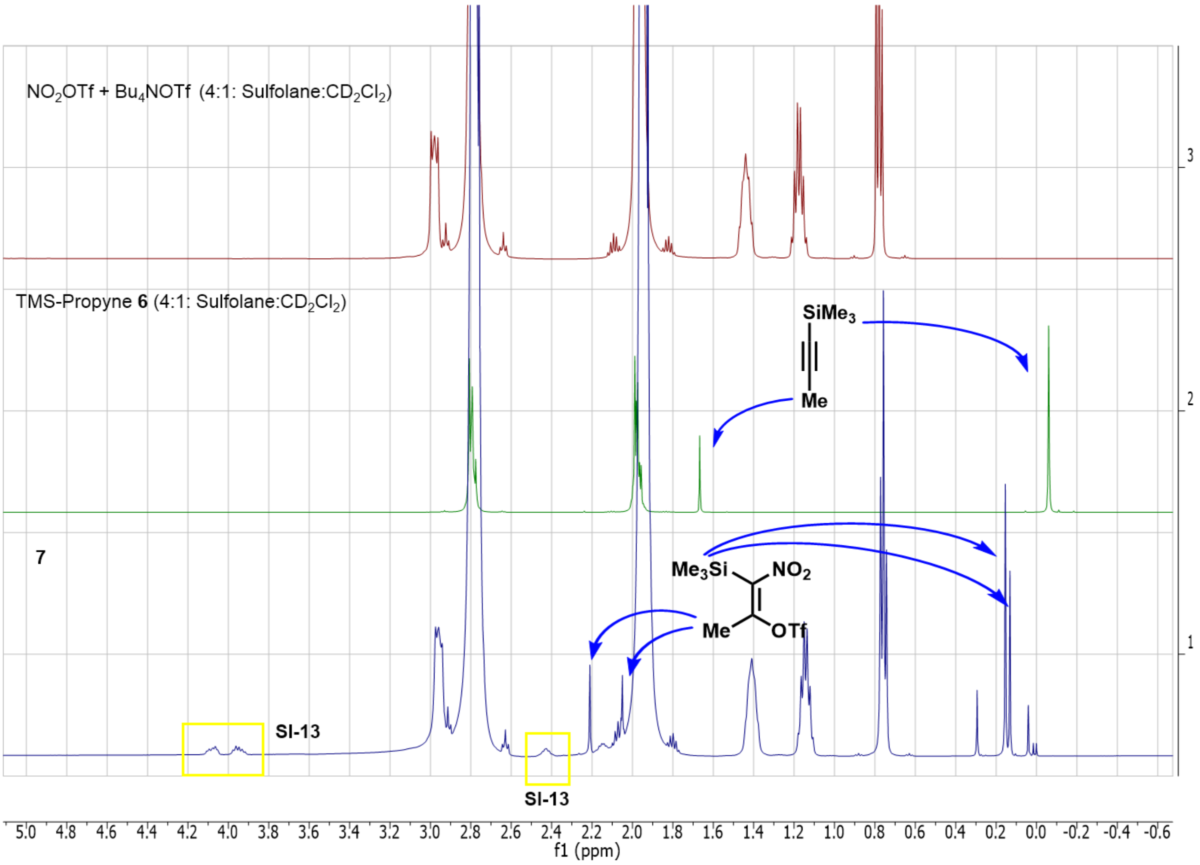
NO2OTf + Bu4NOTf (4:1: Sulfolane:CD2Cl2)
TMS-Propyne 6 (4:1: Sulfolane:CD2Cl2)
SiMe3
Me
7
Me3Si
NO2
Me
OTf
SI-13
SI-13
f1 (ppm)

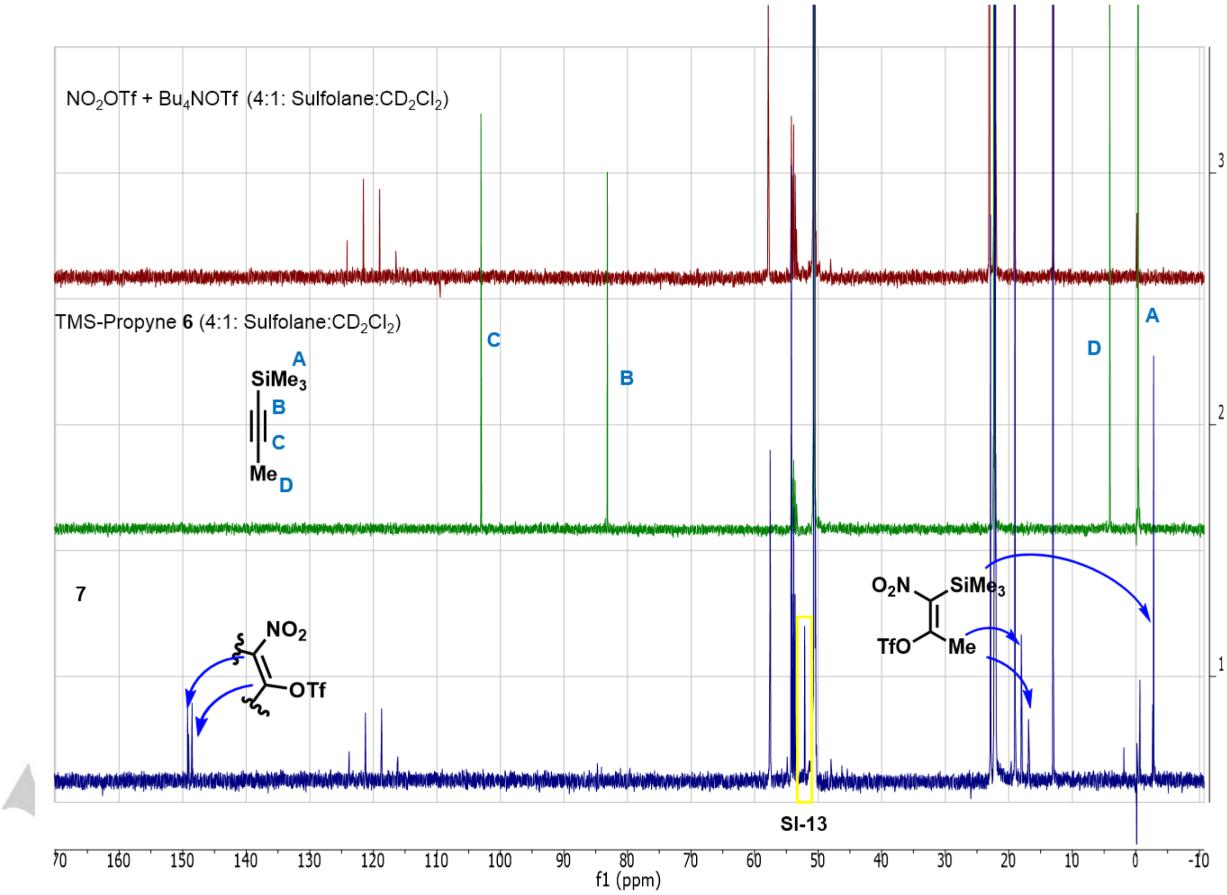
NO2OTf + Bu4NOTf (4:1: Sulfolane:CD2Cl2)
TMS-Propyne 6 (4:1: Sulfolane:CD2Cl2)
A
SiMe3
B
C
Me D
C
B
D
A
7
NO2
OTf
O2N
SiMe3
TfO
Me
SI-13
f1 (ppm)

Figure S2. HSQC Spectrum of **7**.

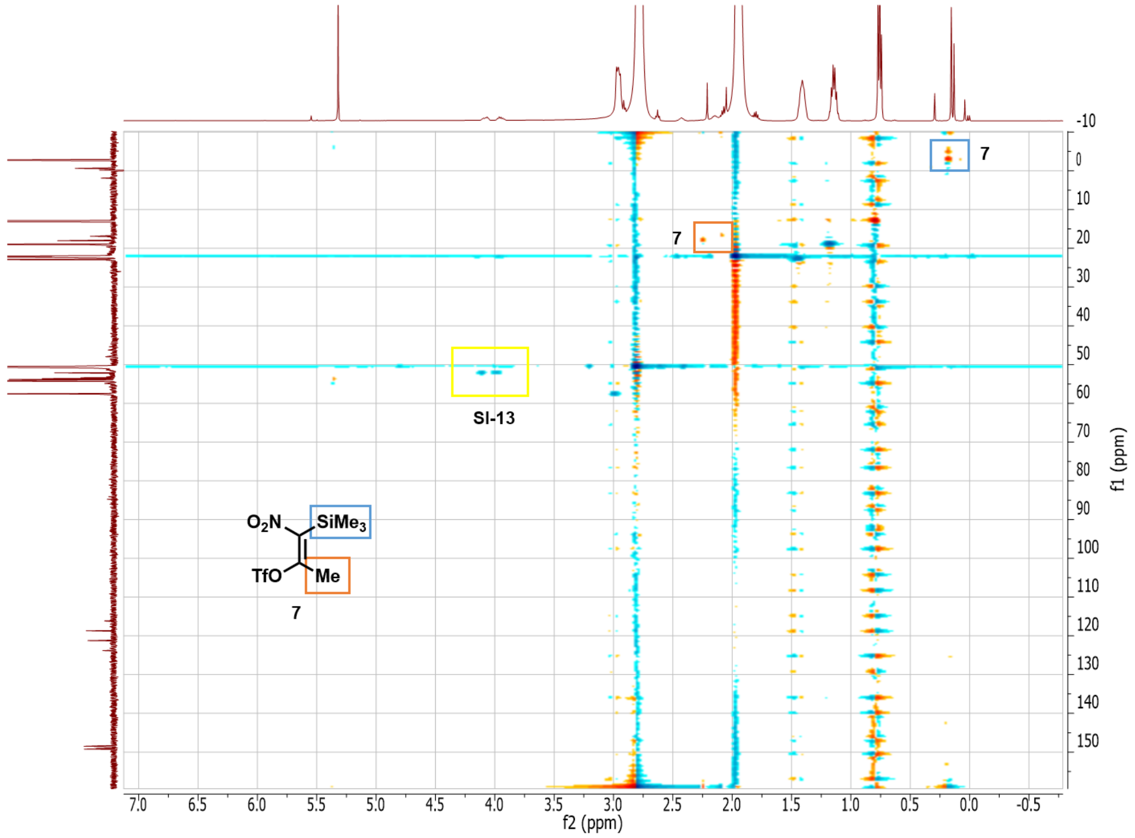

Figure S3. HMBC Spectrum of **7**.

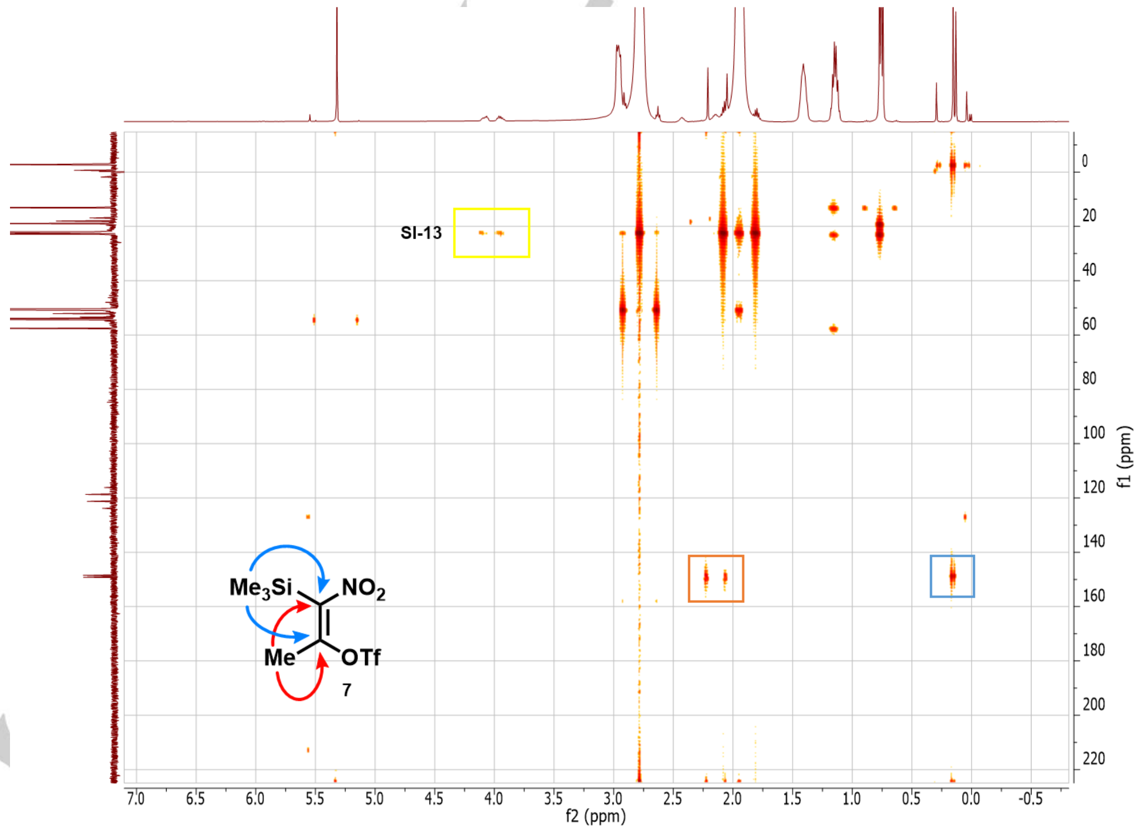

Proposed identity of byproduct **SI-13**: Decomposition of tetrabutylammonium salts

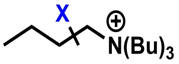

Continuous Flow Experiments

***Figure S4*.** Optimization of the continuous flow nitration and cycloaddition with a nitrone

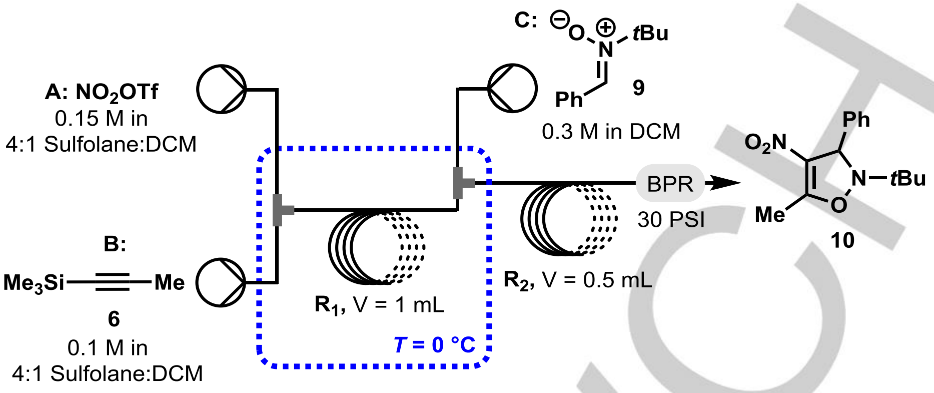

Reactor Setup: Stock solutions were prepared in a glove-box filled with dry nitrogen. Stock **A** was prepared by dissolving 456 mg (1.5 mmol) Bu_4NNO_3 in a mixture of 4:1 sulfolane:DCM in a dry 10 mL volumetric flask. 252 µL Tf_2O were added and the solution was diluted to 10 mL to give a 0.15 M solution of NO_2OTf. Stock **B** was prepared by dissolving 148 µL (1 mmol) tms-propyne in 10 mL 4:1 sulfolane:DCM in a 10 mL volumetric flask. Stock **C** was prepared by dissolving 531 mg (3 mmol) nitrone **8** in 10 mL DCM in a dry volumetric flask. 8 mL of each solution was transferred to 8 mL stainless steel syringes, which were removed from the glove-box and attached to the reactor. Reactor 1 was constructed from 86 5/16” high purity PFA tubing (0.03” ID) giving a volume of 1 mL. Reactor 2 was constructed from 43 3/16” high purity PFA tubing (0.03” ID) giving a volume of 0.5 mL. Reactor 1 was cooled in an ice/water bath. The reactors were joined with PEEK T-mixers in the shown orientation unless varied according to the table below. A BPR set to 30 PSI was affixed to the end of the reactor.
Procedure: Flow rates for each pump were set according to the table below. The system was equilibrated for 2.5 residence times prior to collecting for 1 residence time. The collected material was worked up in the same manner as the batch reactions, and yields of **9** were obtained by ^{1}H NMR using 1,3,5-trimethoxybenzene as an internal standard. Residence times (t_R) are listed for reactor 1.

Entry	Mixer (R_1)	Rate/pump (µL/min)	T_R R_1 (min)	T_R Total (min)	Yield (%)
1	T	20	25	33.3	67
2	T	40	12.5	16.7	71
3	T	80	6.25	8.4	60
4	Y	40	12.5	16.7	64
5	T + static mixer	40	12.5	16.7	73
6	**T + static mixer**	**80**	**6.25**	**8.4**	**72**

WILEY-VCH

Figure S5. Optimization of in-line generation of NO_2OTf

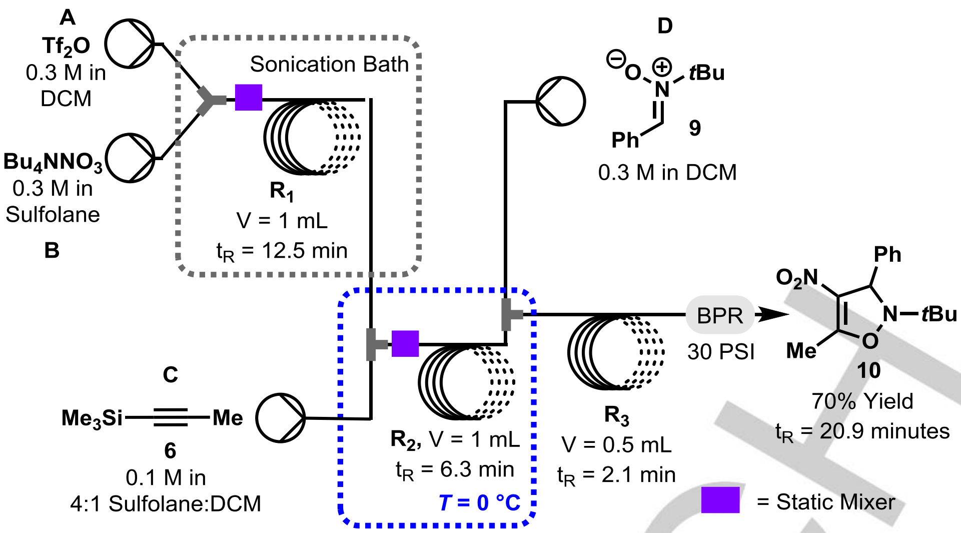

Reactor Setup: Stock solutions were prepared in a glove-box filled with dry nitrogen. Stock **A** was prepared by dissolving 252 µL Tf_2O (1.5 mmol) in 5 mL DCM in a dry 5 mL volumetric flask, giving a 0.3 M solution. Stock **B** was prepared by dissolving 456 mg (1.5 mmol) Bu_4NNO_3 in 5 mL sulfolane in a dry 5 mL volumetric flask. Stock **C** was prepared by dissolving 148 µL (1 mmol) tms-propyne in 10 mL 4:1 sulfolane:DCM in a dry 10 mL volumetric flask. Stock **D** was prepared by dissolving 531 mg (3 mmol) nitrone **8** in 10 mL DCM in a dry volumetric flask. 5 mL of **A** and **B**, along with 8 ml of **C** and **D** were transferred to 8 mL stainless steel syringes, which were removed from the glove-box and attached to the reactor. Reactors 1 and 2 were constructed from 86 5/16" high purity PFA tubing (0.03" ID) giving a volume of 1 mL. Reactor 3 was constructed from 43 3/16" high purity PFA tubing (0.03" ID) giving a volume of 0.5 mL. Reactor 2 was cooled in an ice/water bath. The reactors were joined with PEEK Y- or T-mixers in the shown orientation, with a static mixer placed after the Y-mixer in reactor 1 and after the T-mixer in reactor 2. For entry 2 reactor 1 was placed in a sonication bath. A BPR set to 30 PSI was affixed to the end of the reactor.
Procedure: Stocks **A** and **B** were pumped at a rate of 40 µL/min each, giving a residence time of 12.5 min for reactor 1. Stocks **C** and **D** were pumped at a rate of 80 µL/min each, giving a residence time of 6.3 minutes for reactor 2 and 2.1 minutes for reactor 3, and an overall residence time of 20.9 minutes. The system was equilibrated for 2.5 residence times prior to collecting for 1 residence time for entry 1 and 4 residence times for entry 2. The collected material was worked up in the same manner as the batch reactions, and yields of **9** were obtained by ^{1}H NMR using 1,3,5-trimethoxybenzene as an internal standard.

Entry	Mixing (Reactor 1)	Yield (%)
1	Y-mixer + static mixer	36
**2**	**Y-mixer + static mixer + sonication**	**70**

References

[1] Barber, J. S.; Styduhar, E. D.; Pham, H. V.; McMahon, T. C.; Houk, K. N.; Garg, N. K. *J. Am. Chem. Soc.* **2016**, *138* (8), 2512–2515.

WILEY-VCH

^{1}H and ^{13}C NMR Spectra

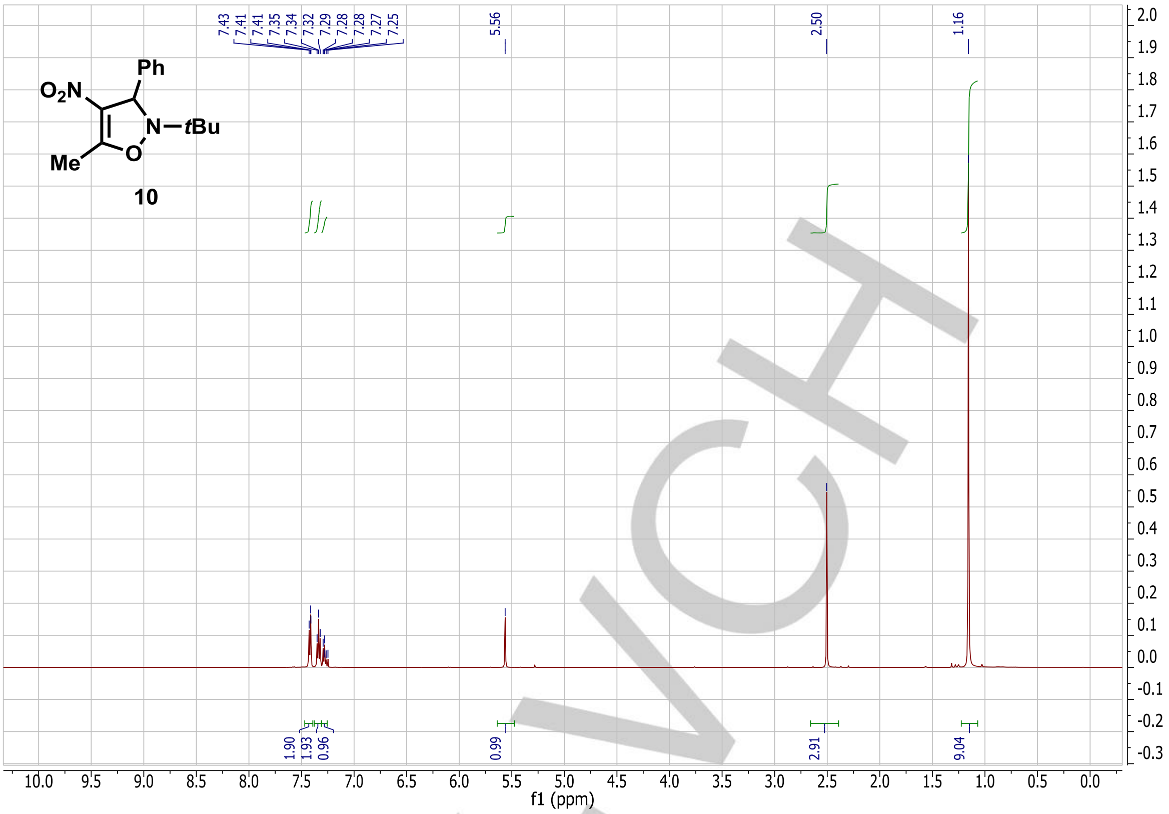

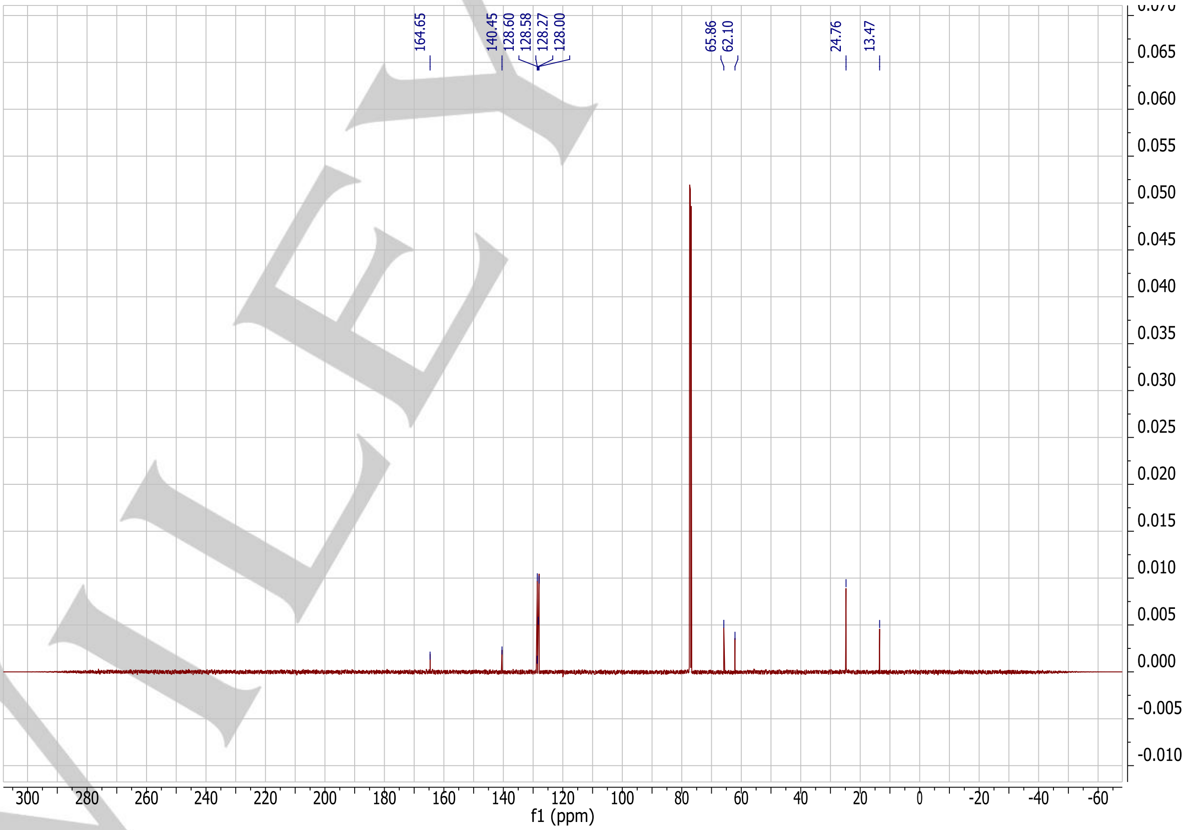

WILEY-VCH

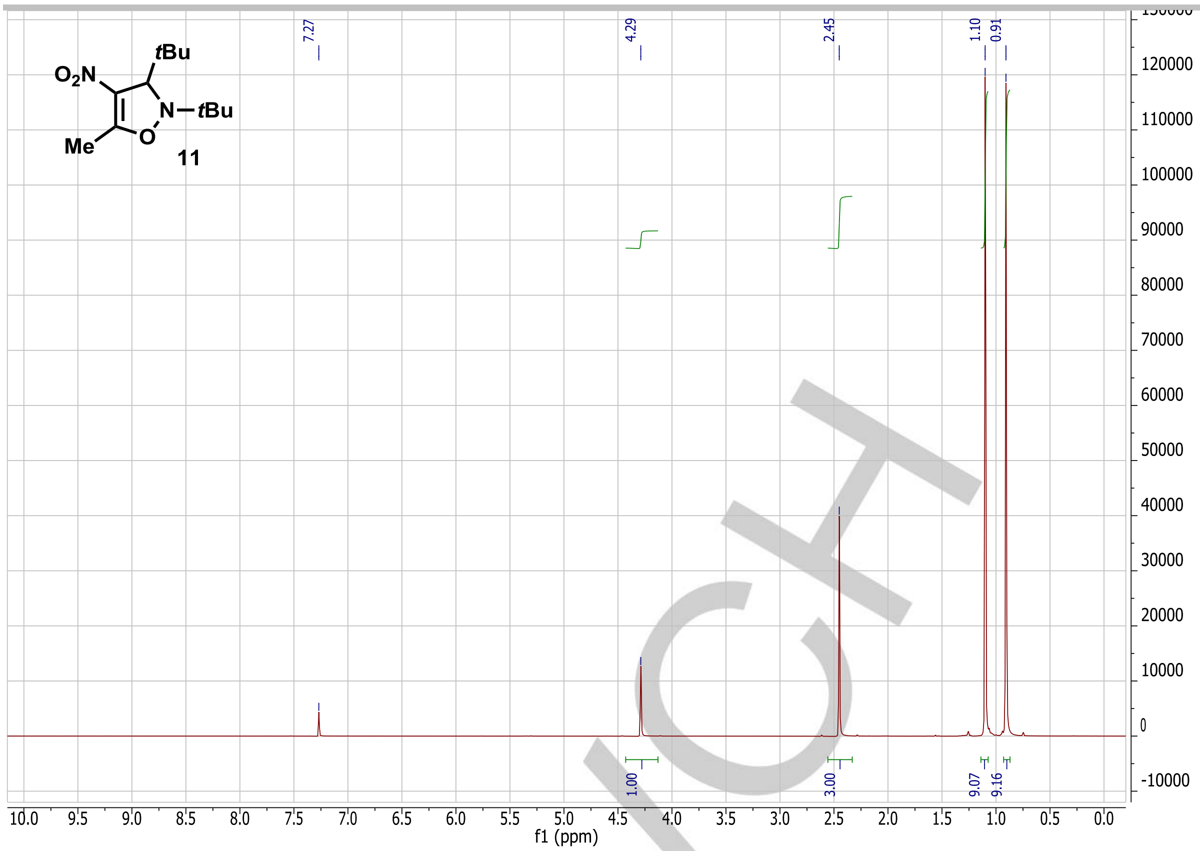
11
7.27
4.29
2.45
1.10
0.91
1.00
3.00
9.07
9.16
f1 (ppm)

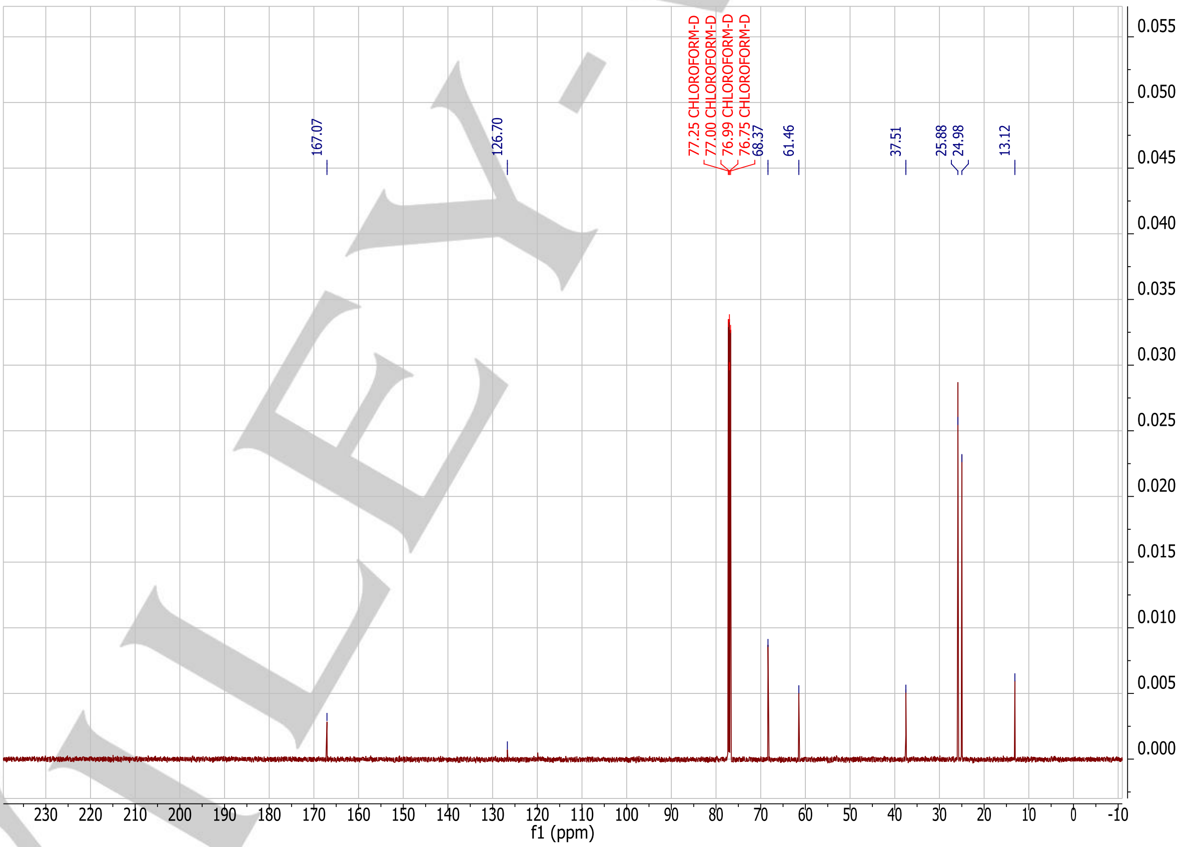
167.07
126.70
77.25 CHLOROFORM-D
77.00 CHLOROFORM-D
76.99 CHLOROFORM-D
76.75 CHLOROFORM-D
68.37
61.46
37.51
25.88
24.98
13.12
f1 (ppm)

WILEY-VCH

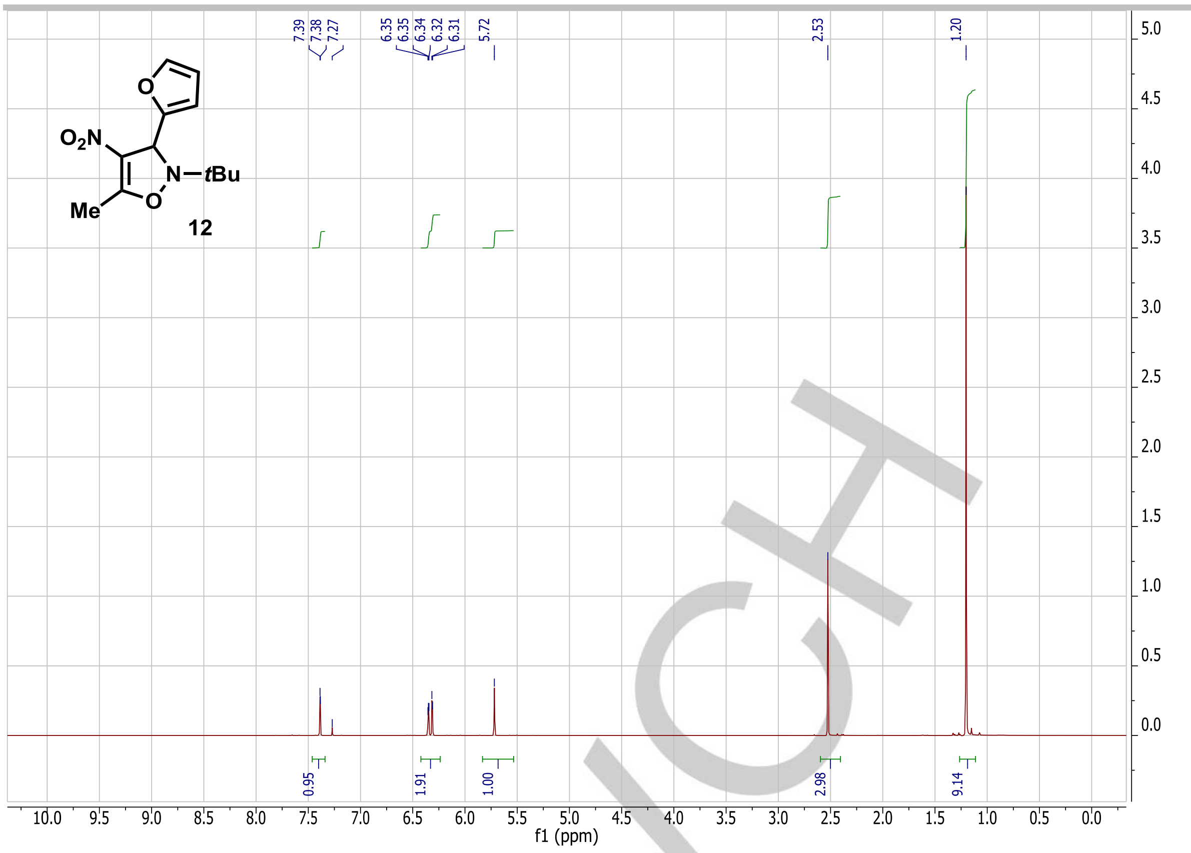
7.39
7.38
7.27
6.35
6.35
6.34
6.32
6.31
5.72
2.53
1.20
O_2N
N—tBu
Me
O
O
12
0.95
1.91
1.00
2.98
9.14
f1 (ppm)

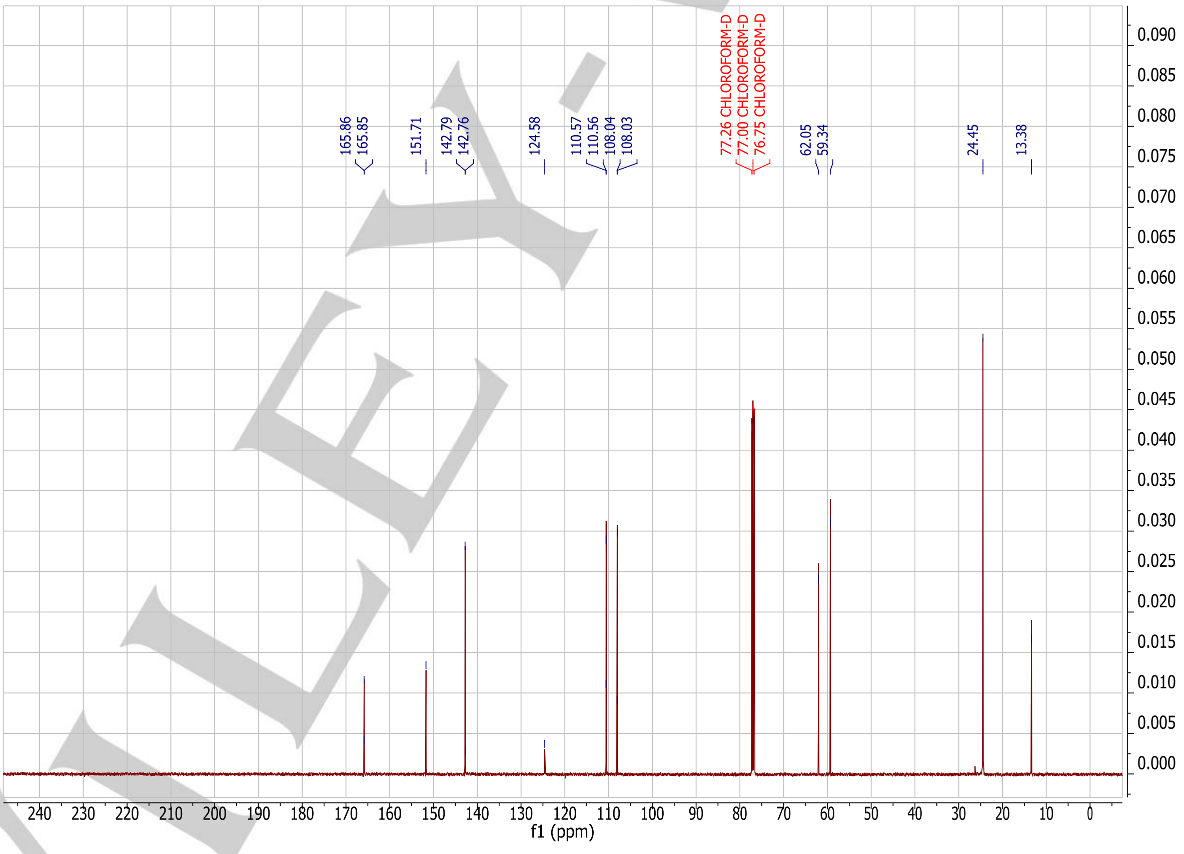
165.86
165.85
151.71
142.79
142.76
124.58
110.57
110.56
108.04
108.03
77.26 CHLOROFORM-D
77.00 CHLOROFORM-D
76.75 CHLOROFORM-D
62.05
59.34
24.45
13.38
f1 (ppm)

WILEY-VCH

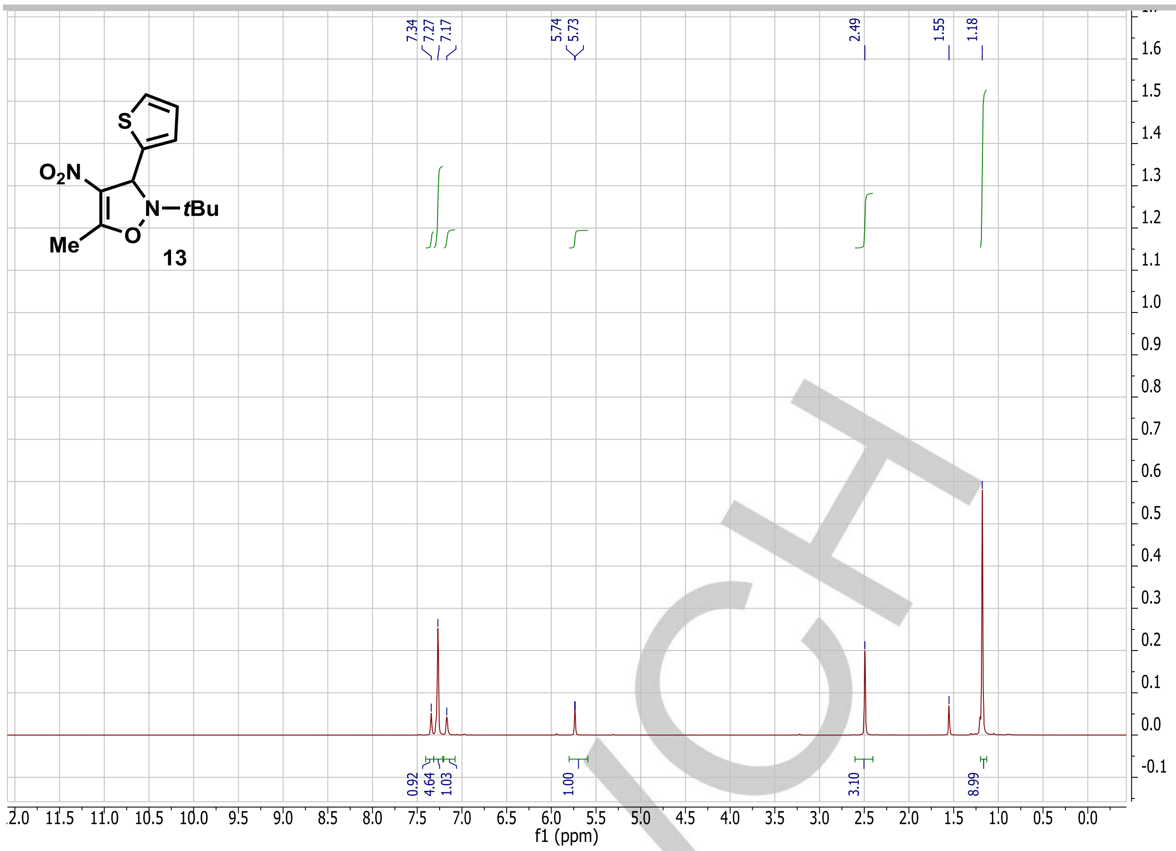
7.34
7.27
7.17
5.74
5.73
2.49
1.55
1.18
O2N
S
N−tBu
Me
O
13
0.92
4.64
1.03
1.00
3.10
8.99
f1 (ppm)

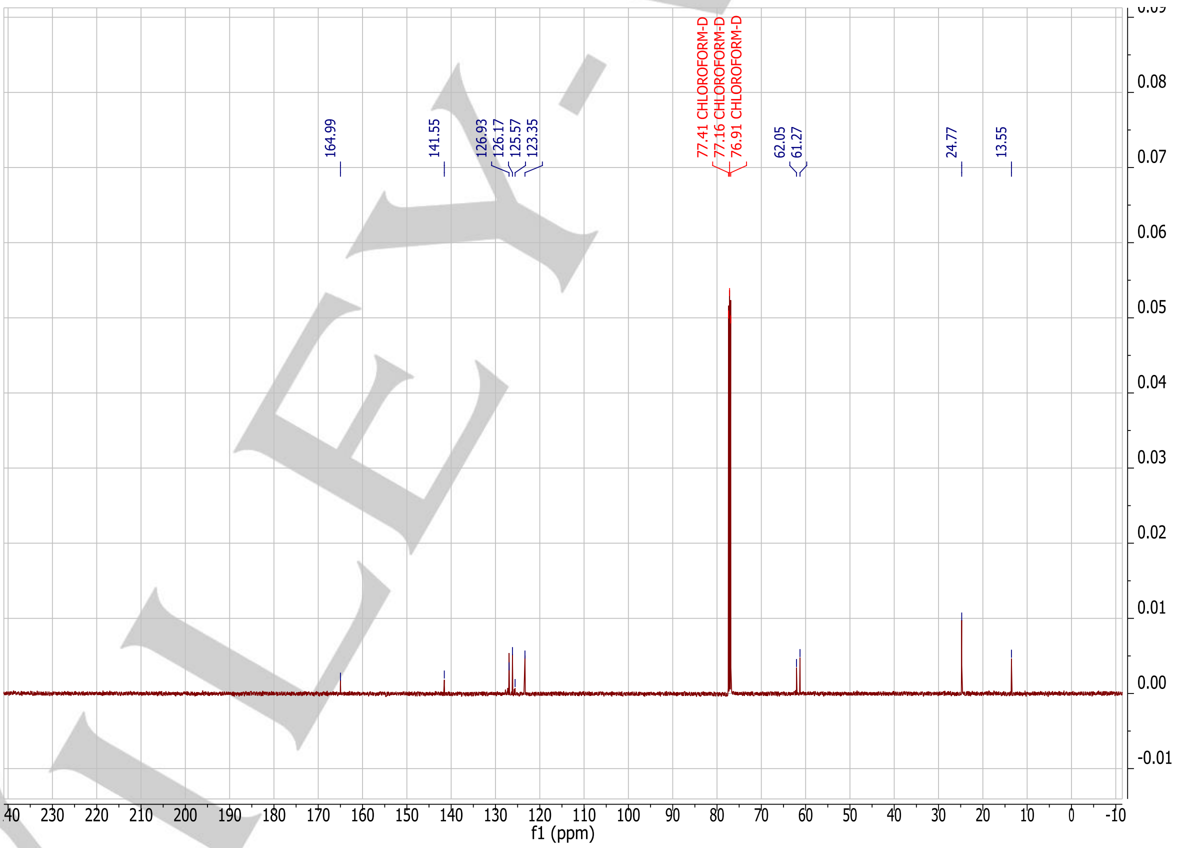
164.99
141.55
126.93
126.17
125.57
123.35
77.41 CHLOROFORM-D
77.16 CHLOROFORM-D
76.91 CHLOROFORM-D
62.05
61.27
24.77
13.55
f1 (ppm)

WILEY-VCH

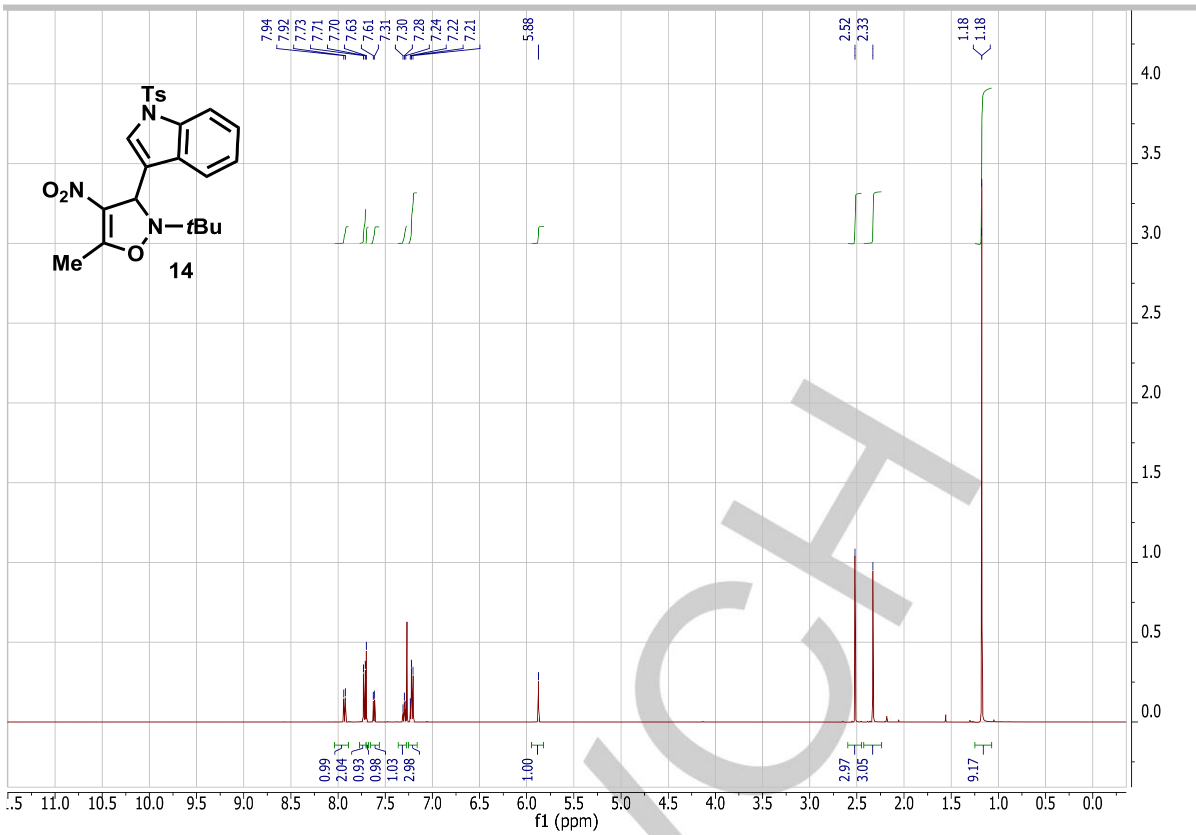

Ts
N
O2N
N—tBu
Me
O
14
7.94 7.92 7.73 7.71 7.70 7.63 7.61 7.31 7.30 7.28 7.24 7.22 7.21
5.88
2.52 2.33
1.18 1.18
0.99 2.04 0.93 0.98 1.03 2.98 1.00 2.97 3.05 9.17
f1 (ppm)

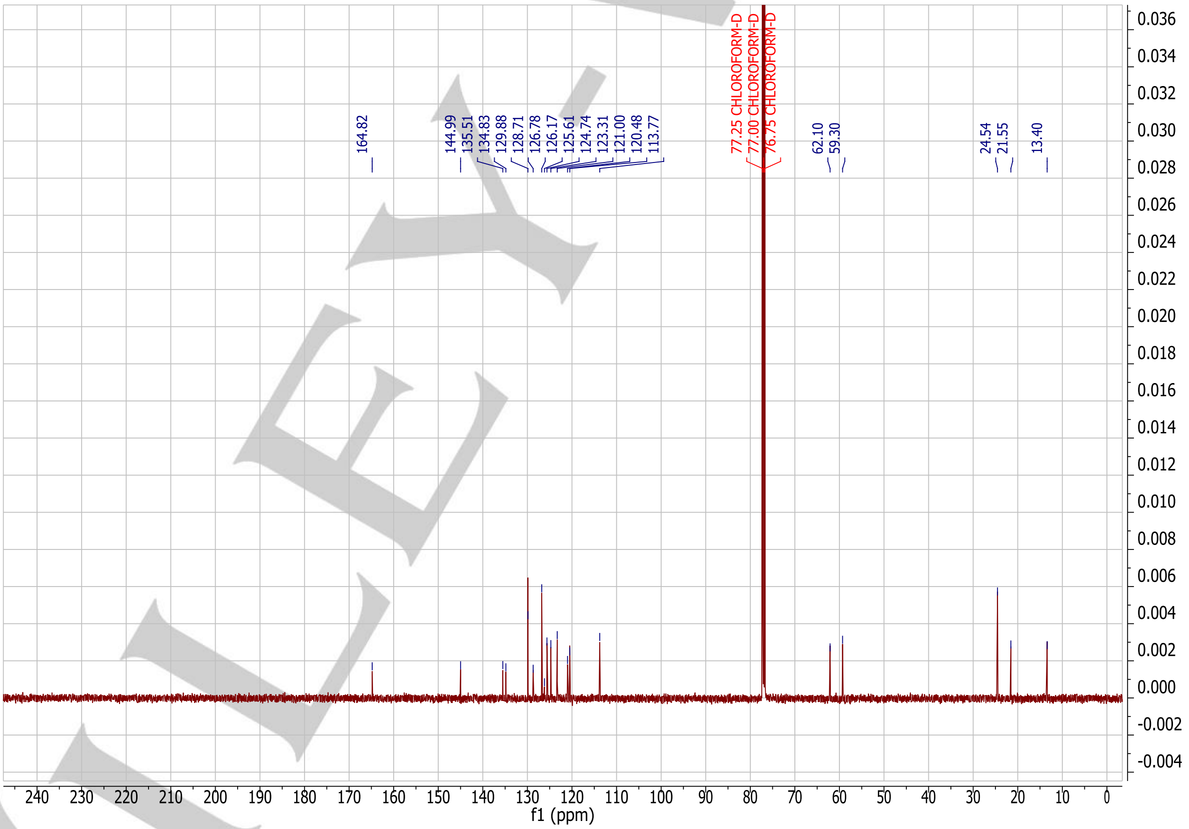

164.82
144.99 135.51 134.83 129.88 128.71 126.78 126.17 125.61 124.74 123.31 121.00 120.48 113.77
77.25 CHLOROFORM-D
77.00 CHLOROFORM-D
76.75 CHLOROFORM-D
62.10 59.30
24.54 21.55
13.40
f1 (ppm)

WILEY-VCH

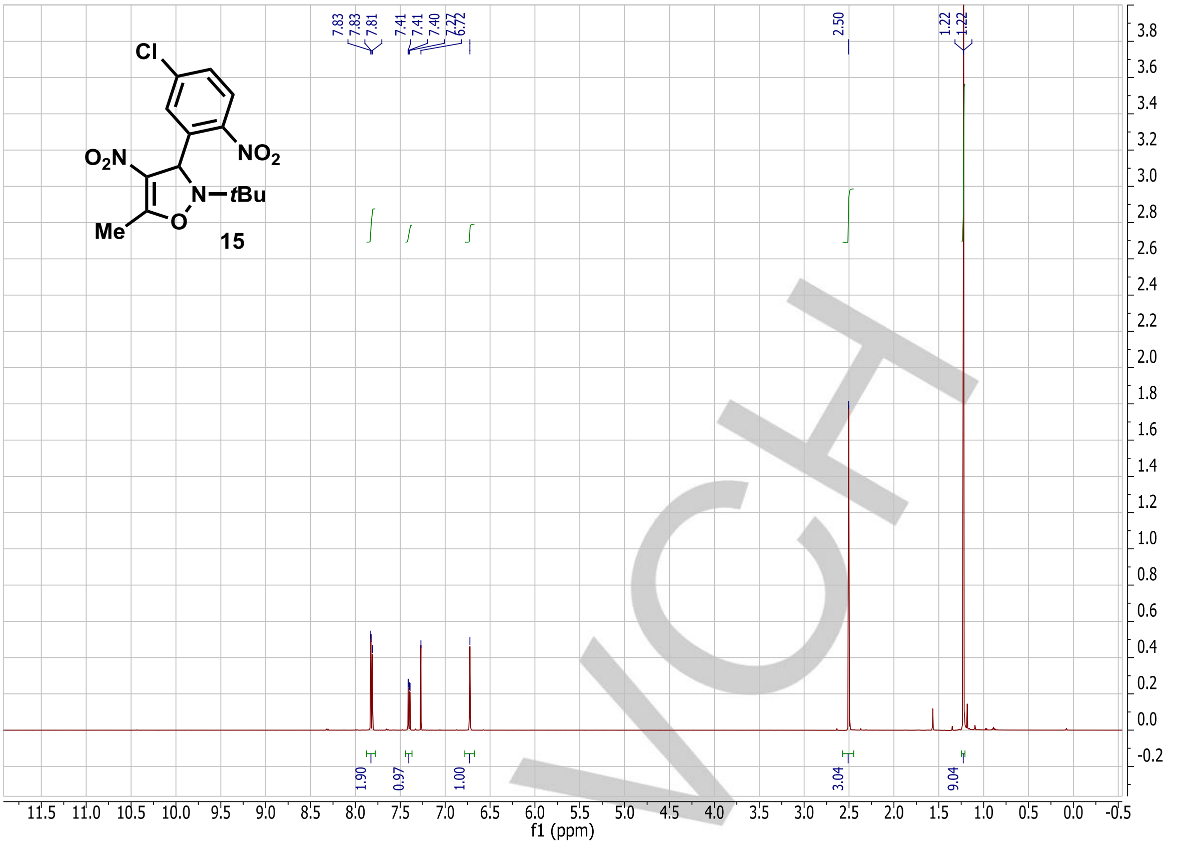
Cl
NO_2
O_2N
N—tBu
O
Me
15
7.83
7.83
7.81
7.41
7.41
7.40
7.27
6.72
2.50
1.22
1.22
1.90
0.97
1.00
3.04
9.04
f1 (ppm)

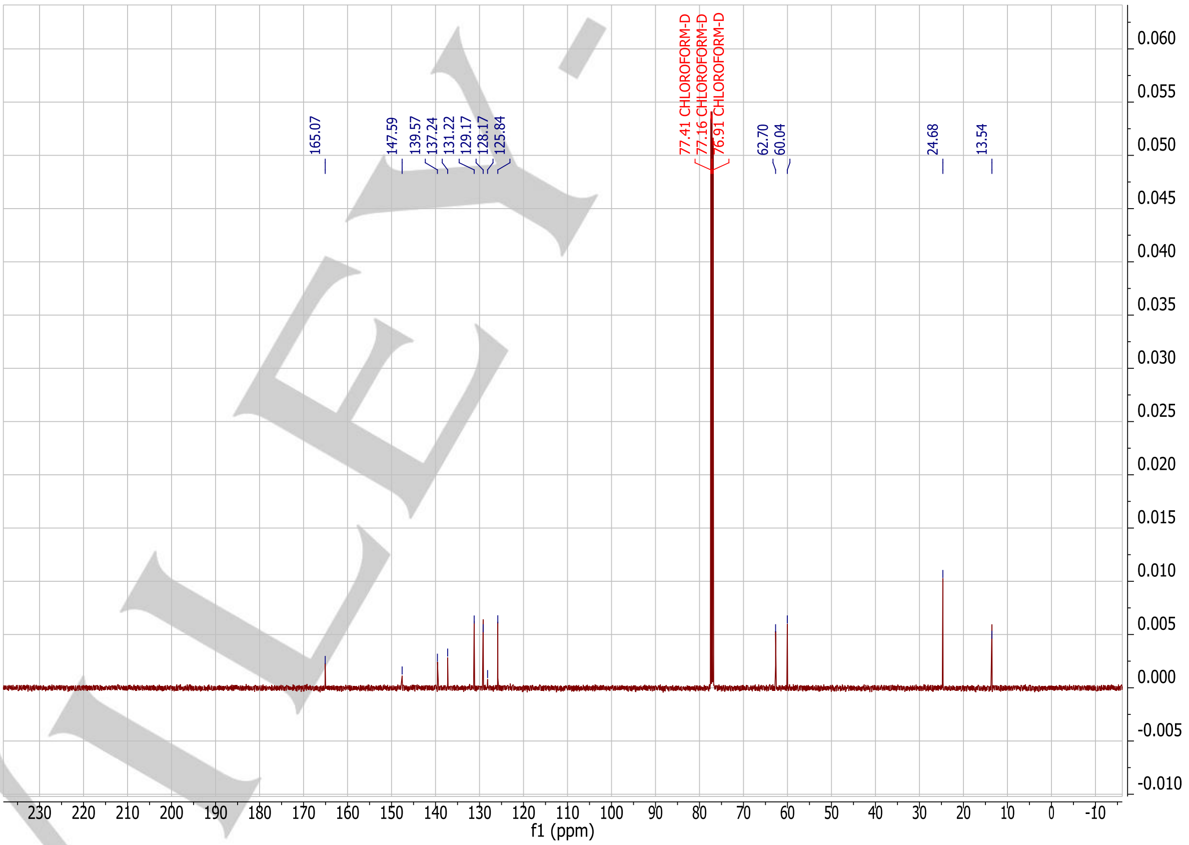
165.07
147.59
139.57
137.24
131.22
129.17
128.17
125.84
77.41 CHLOROFORM-D
77.16 CHLOROFORM-D
76.91 CHLOROFORM-D
62.70
60.04
24.68
13.54
f1 (ppm)

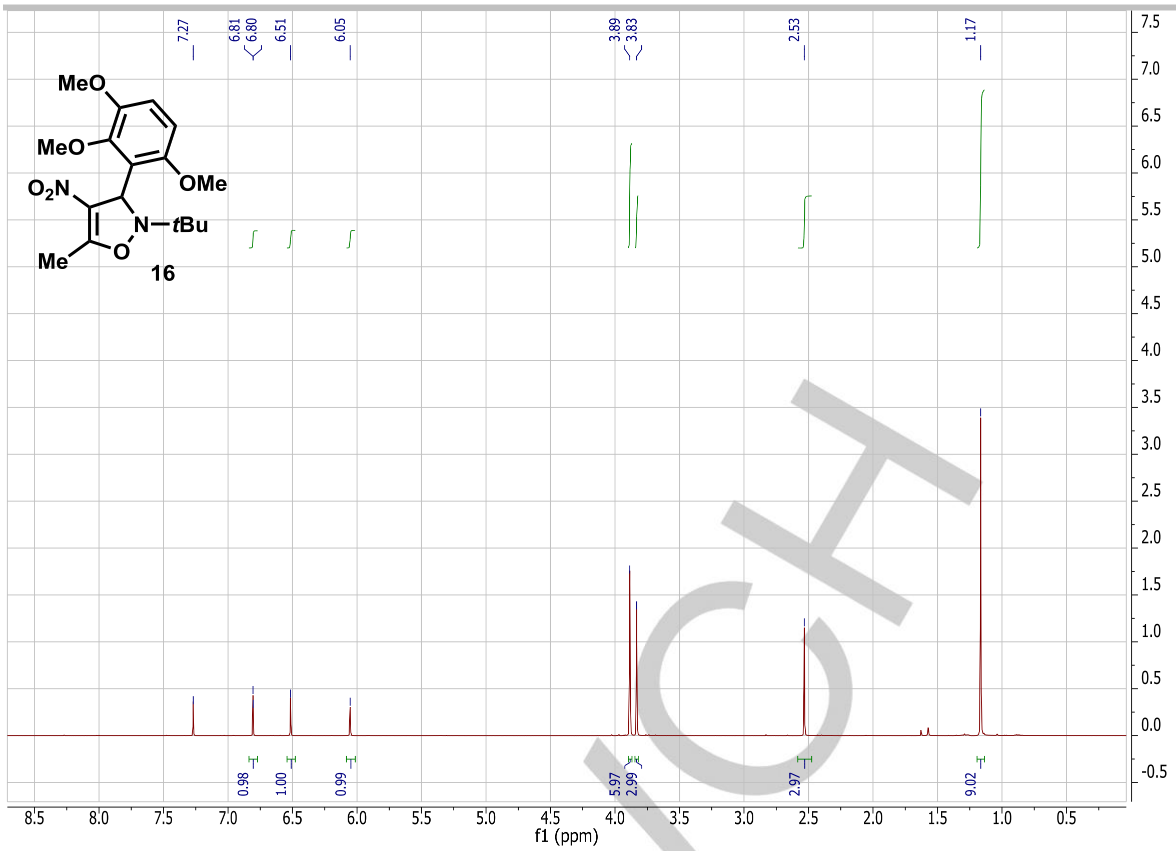

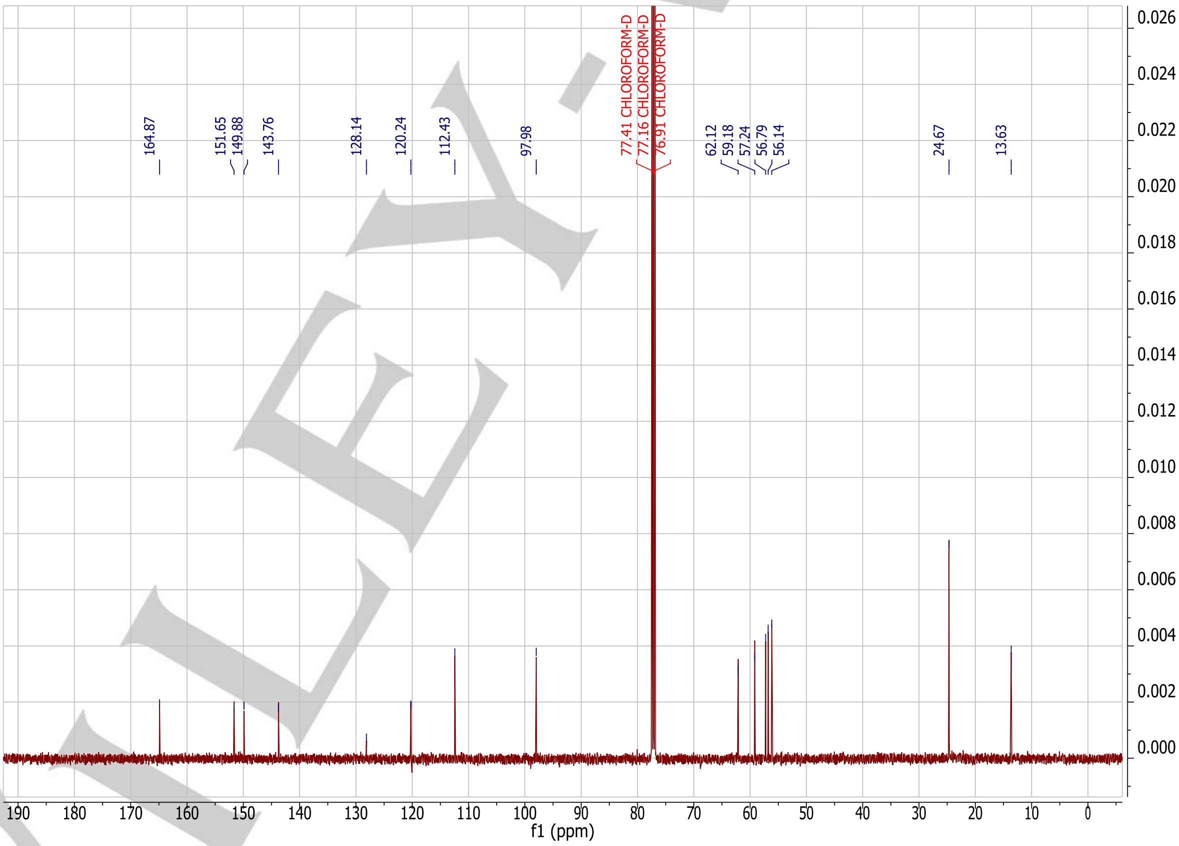

WILEY-VCH

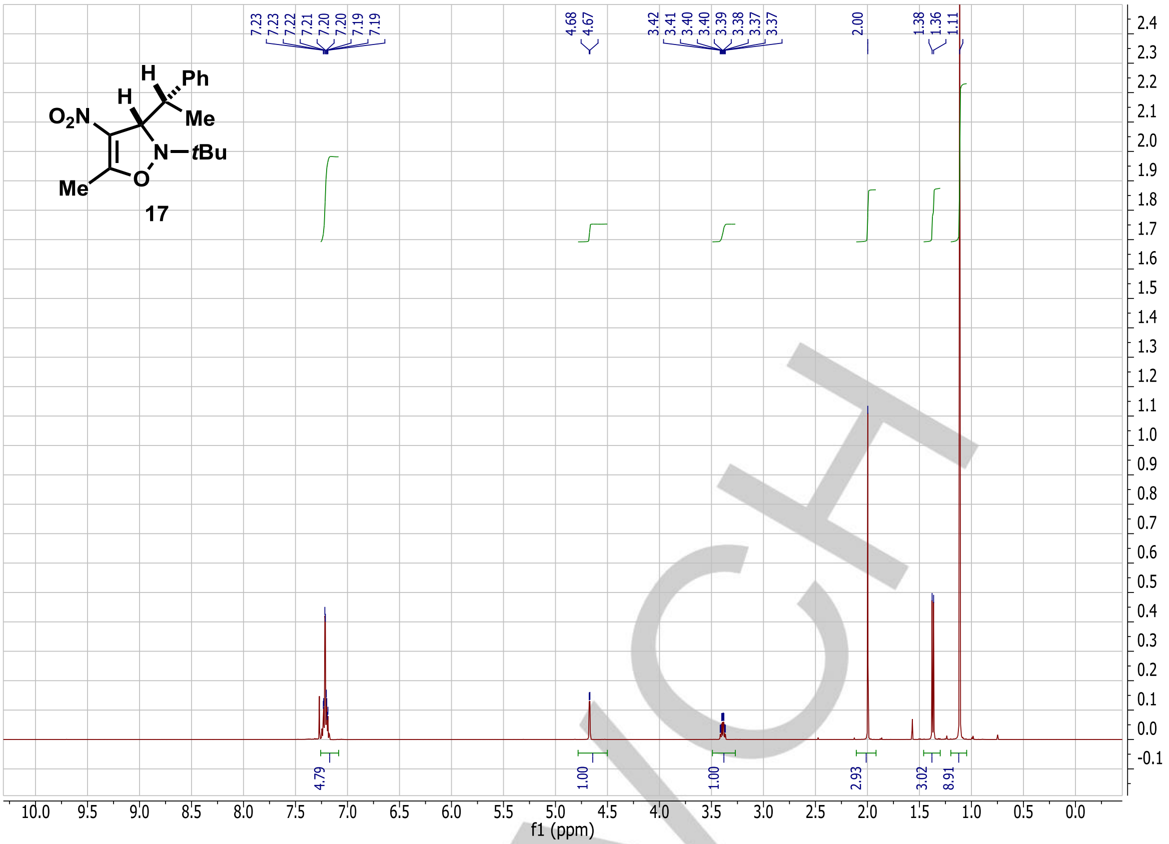
17
7.23
7.23
7.22
7.21
7.20
7.20
7.19
7.19
4.68
4.67
3.42
3.41
3.40
3.40
3.39
3.38
3.37
3.37
2.00
1.38
1.36
1.11
4.79
1.00
1.00
2.93
3.02
8.91
f1 (ppm)

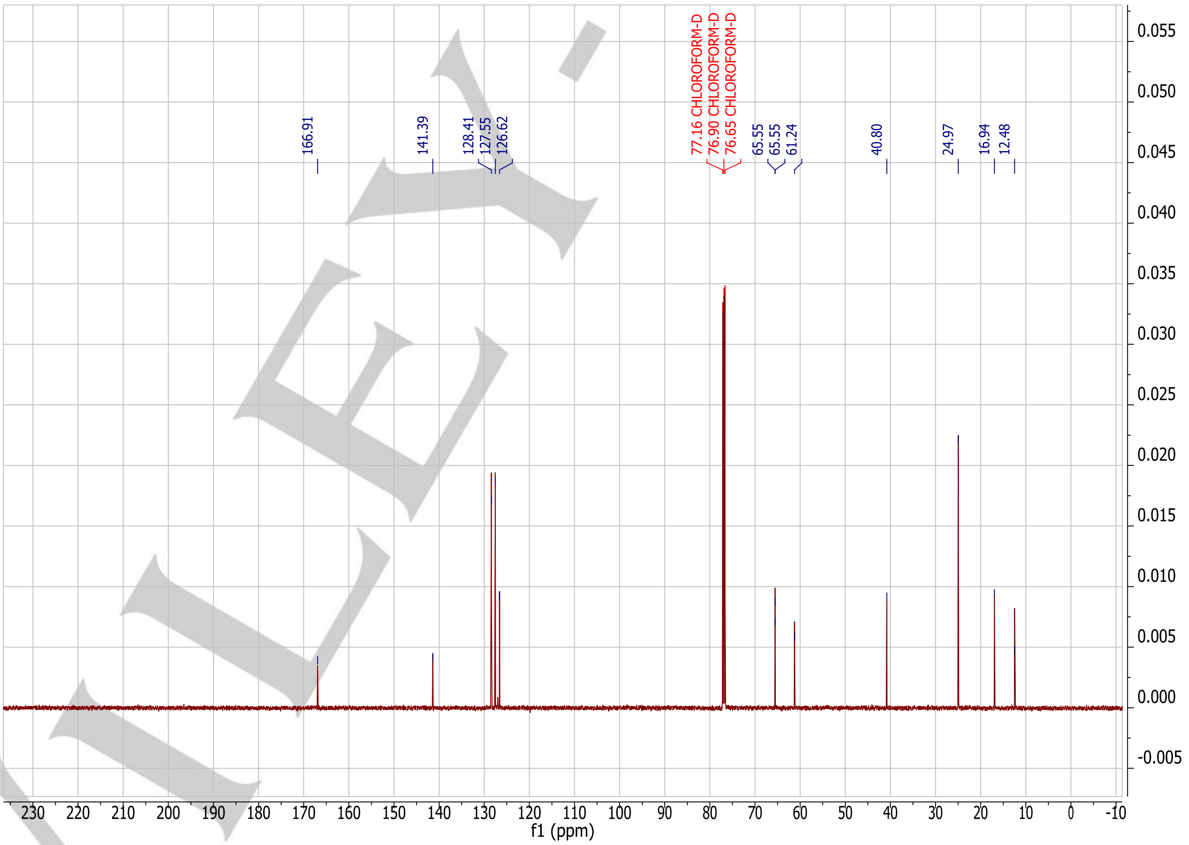
166.91
141.39
128.41
127.55
126.62
77.16 CHLOROFORM-D
76.90 CHLOROFORM-D
76.65 CHLOROFORM-D
65.55
65.55
61.24
40.80
24.97
16.94
12.48
f1 (ppm)

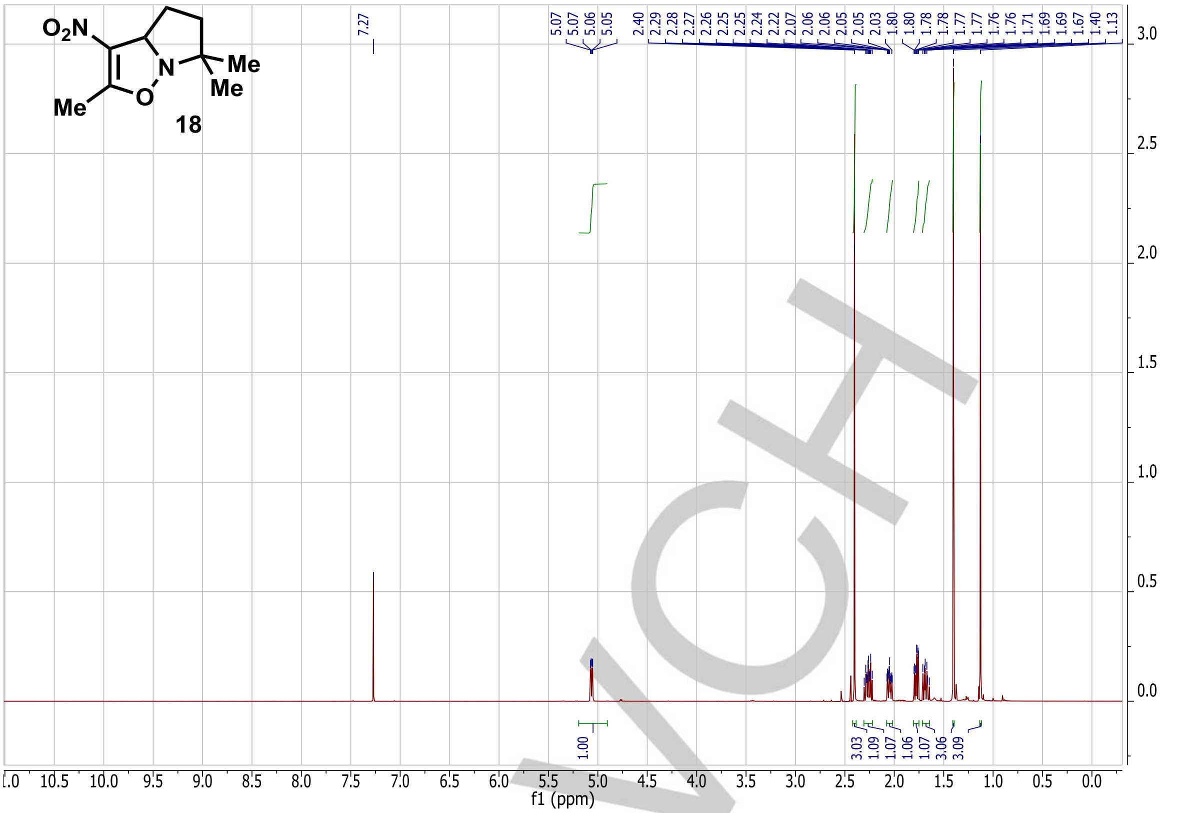

O2N
N
O
Me
Me
Me
18
7.27
5.07
5.07
5.06
5.05
2.40
2.29
2.28
2.27
2.26
2.25
2.25
2.24
2.22
2.07
2.06
2.06
2.05
2.05
2.03
1.80
1.80
1.78
1.78
1.77
1.77
1.76
1.71
1.69
1.67
1.40
1.13
1.00
3.03
1.09
1.07
1.06
1.07
3.06
3.09
f1 (ppm)

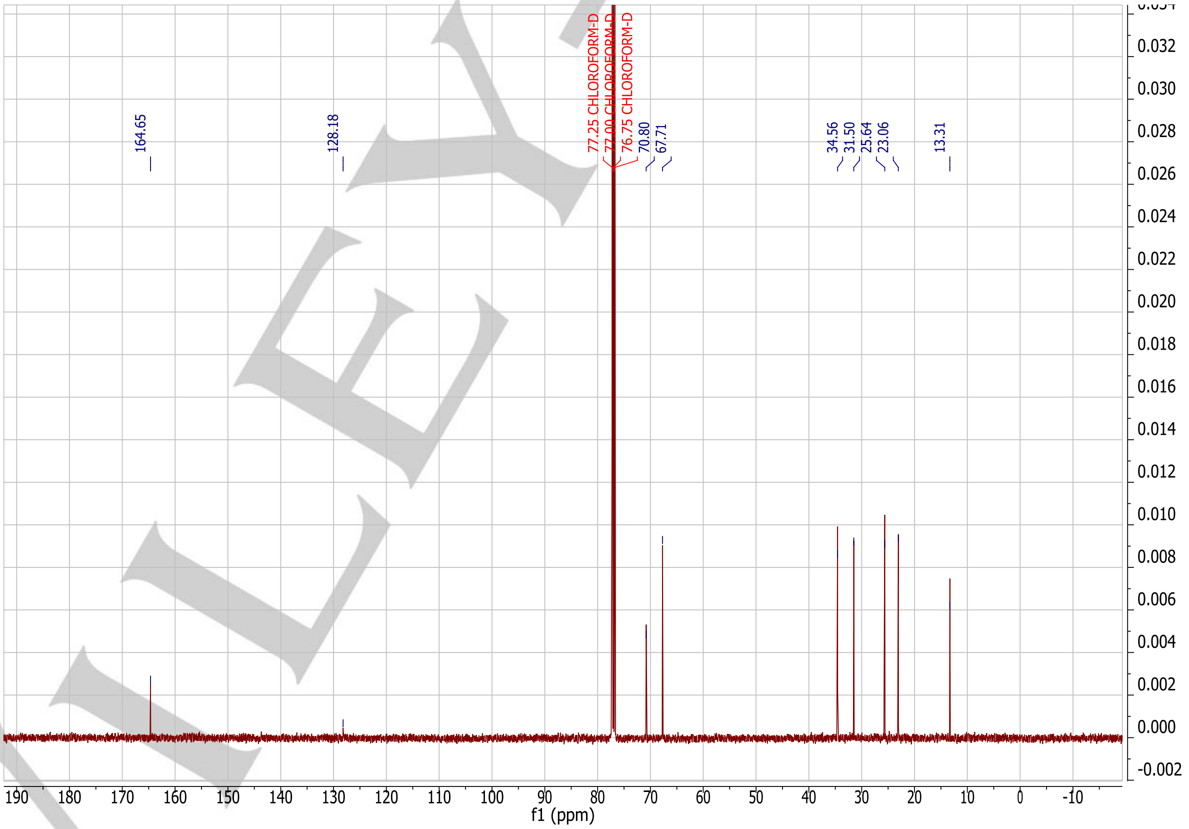

164.65
128.18
77.25 CHLOROFORM-D
77.00 CHLOROFORM-D
76.75 CHLOROFORM-D
70.80
67.71
34.56
31.50
25.64
23.06
13.31
f1 (ppm)

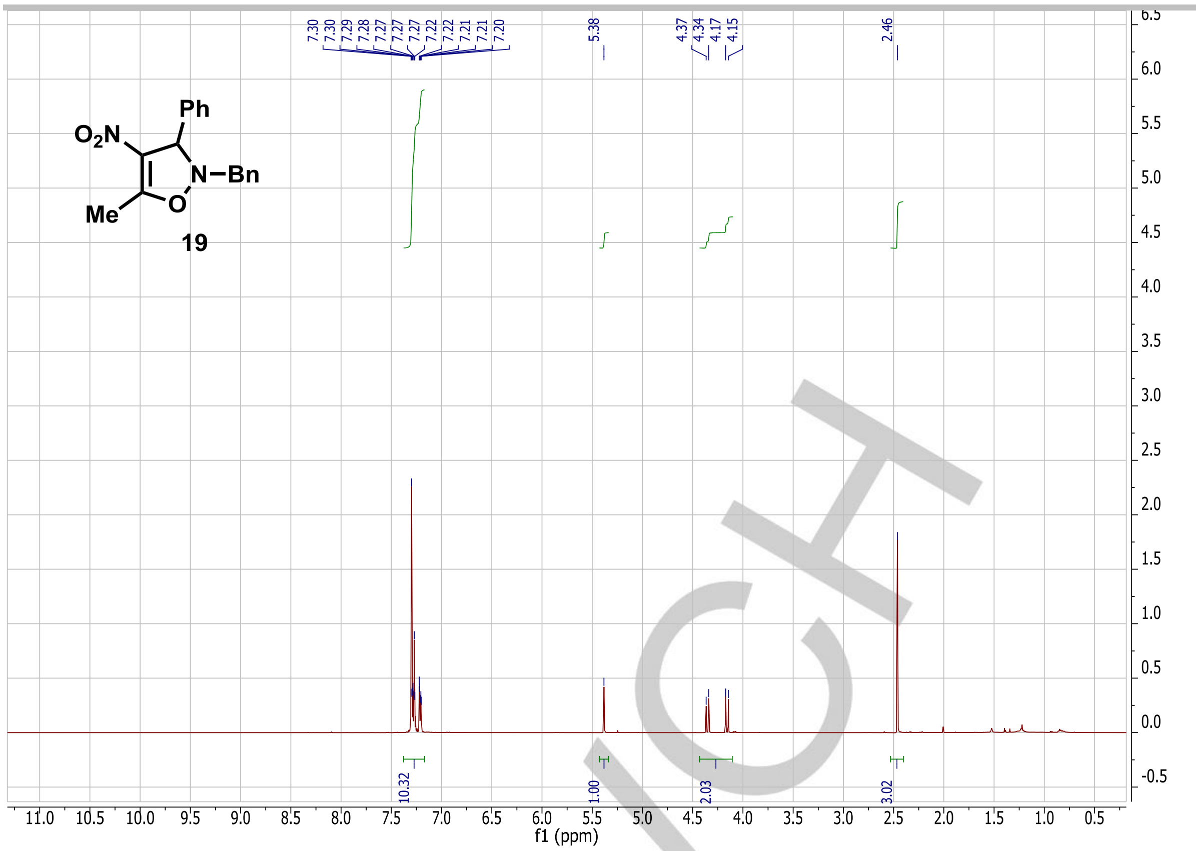
7.30
7.30
7.29
7.28
7.27
7.27
7.27
7.22
7.22
7.21
7.21
7.20
5.38
4.37
4.34
4.17
4.15
2.46
O_2N
Ph
N−Bn
O
Me
19
10.32
1.00
2.03
3.02
f1 (ppm)

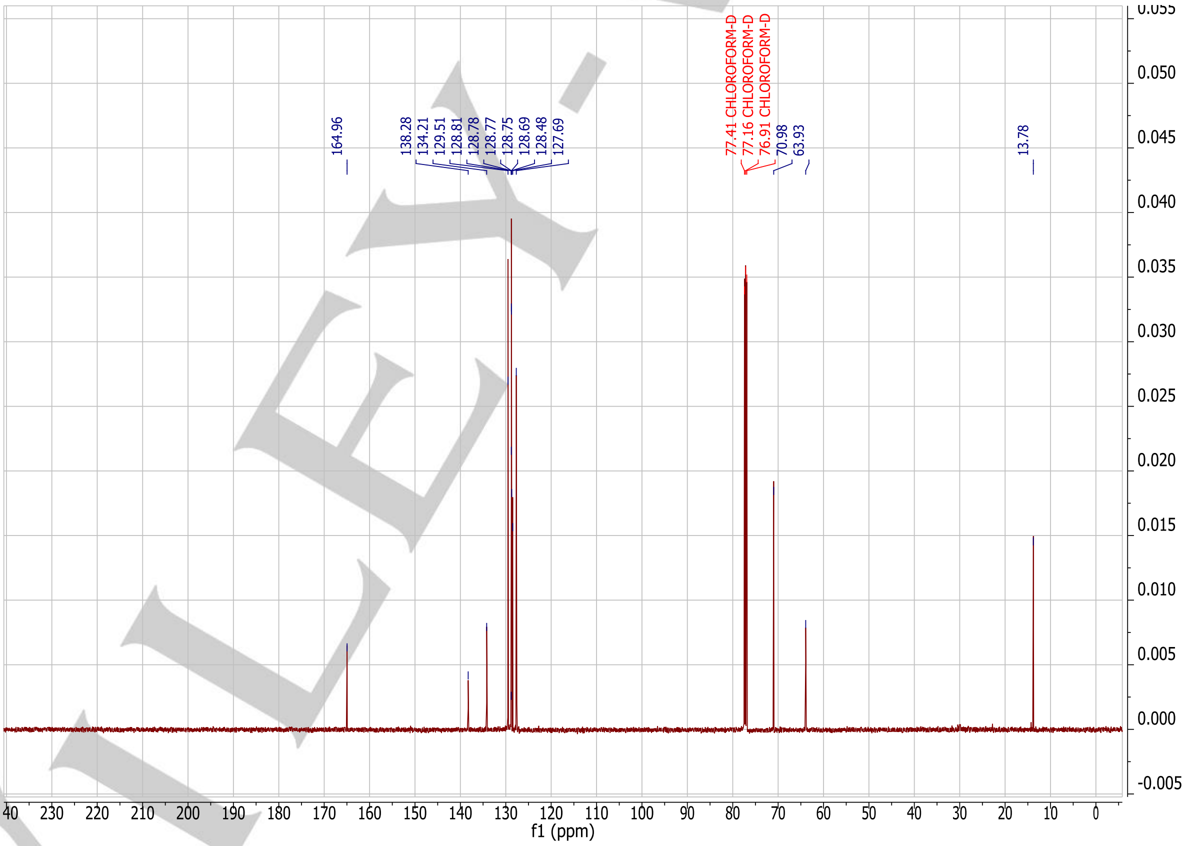
164.96
138.28
134.21
129.51
128.81
128.78
128.77
128.75
128.69
128.48
127.69
77.41 CHLOROFORM-D
77.16 CHLOROFORM-D
76.91 CHLOROFORM-D
70.98
63.93
13.78
f1 (ppm)

WILEY-VCH

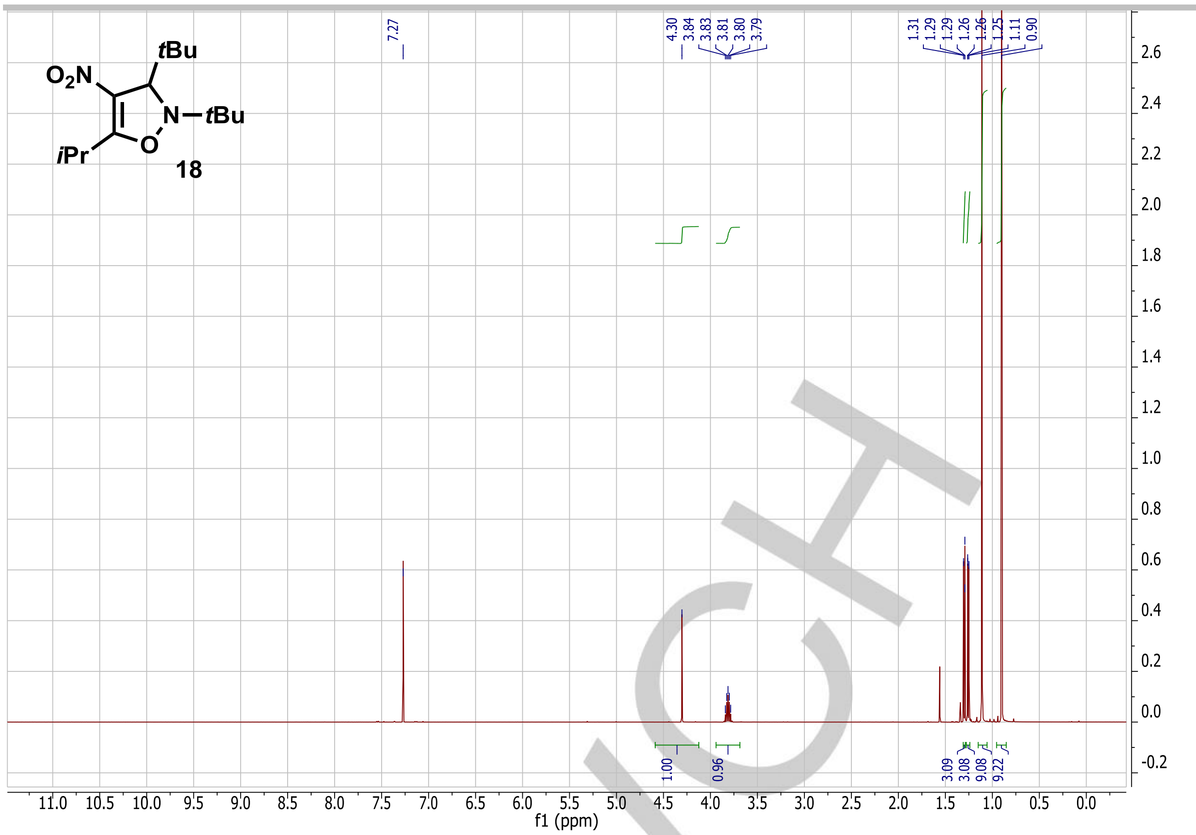
O2N
tBu
N–tBu
iPr
O
18
7.27
4.30
3.84
3.83
3.81
3.80
3.79
1.31
1.29
1.29
1.26
1.26
1.25
1.11
0.90
1.00
0.96
3.09
3.08
9.08
9.22
f1 (ppm)

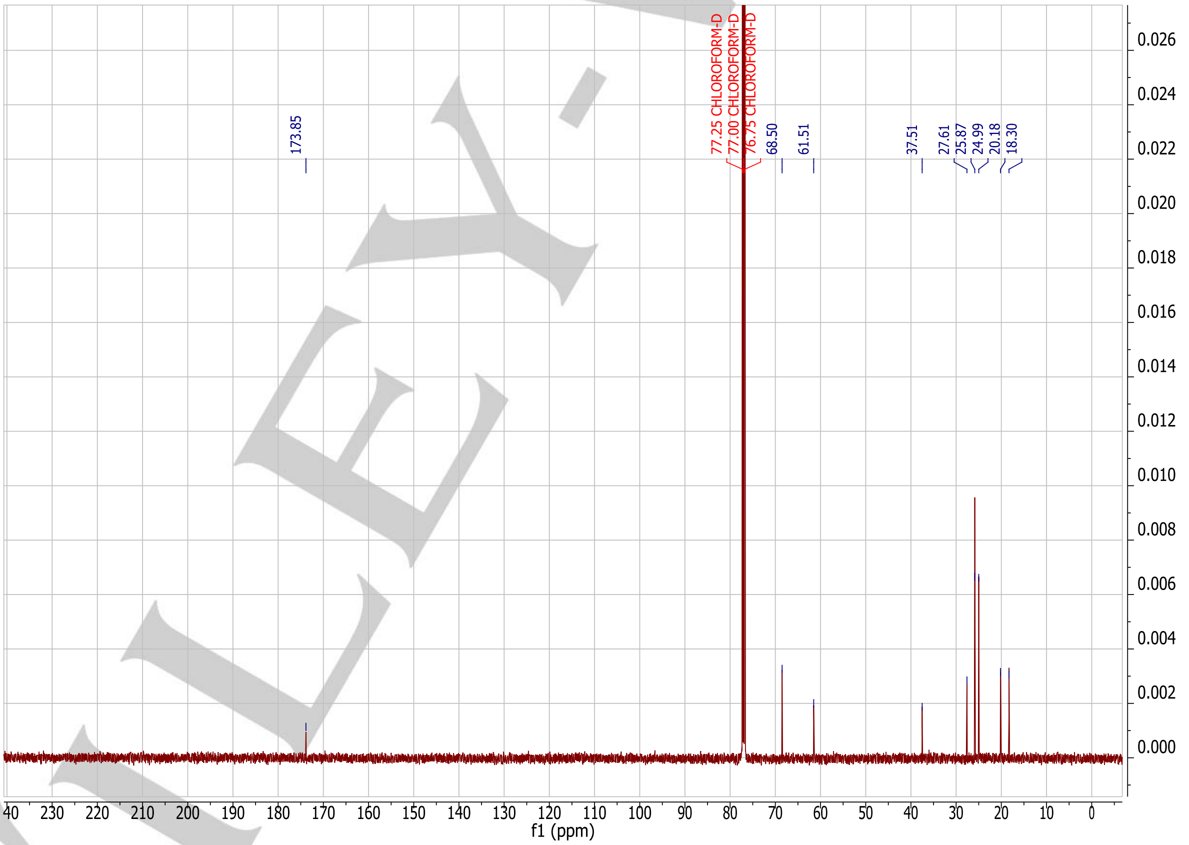
173.85
77.25 CHLOROFORM-D
77.00 CHLOROFORM-D
76.75 CHLOROFORM-D
68.50
61.51
37.51
27.61
25.87
24.99
20.18
18.30
f1 (ppm)

WILEY-VCH

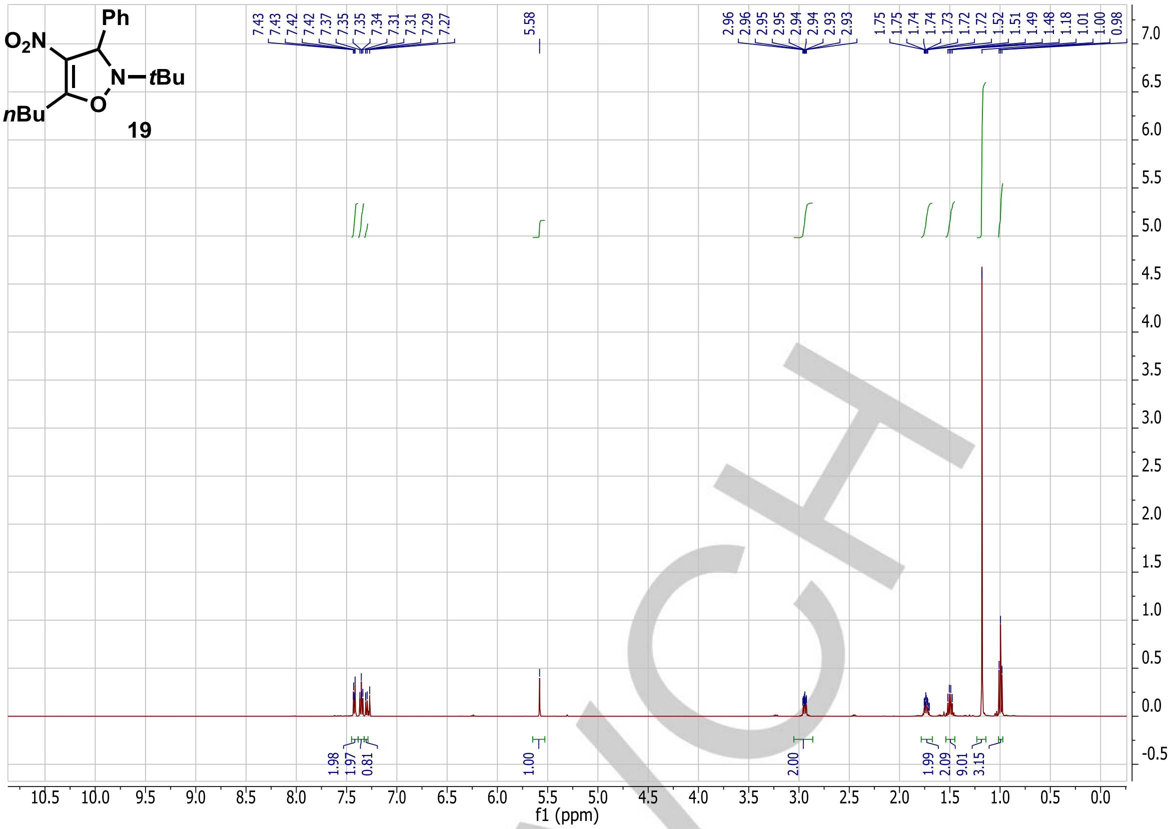

19

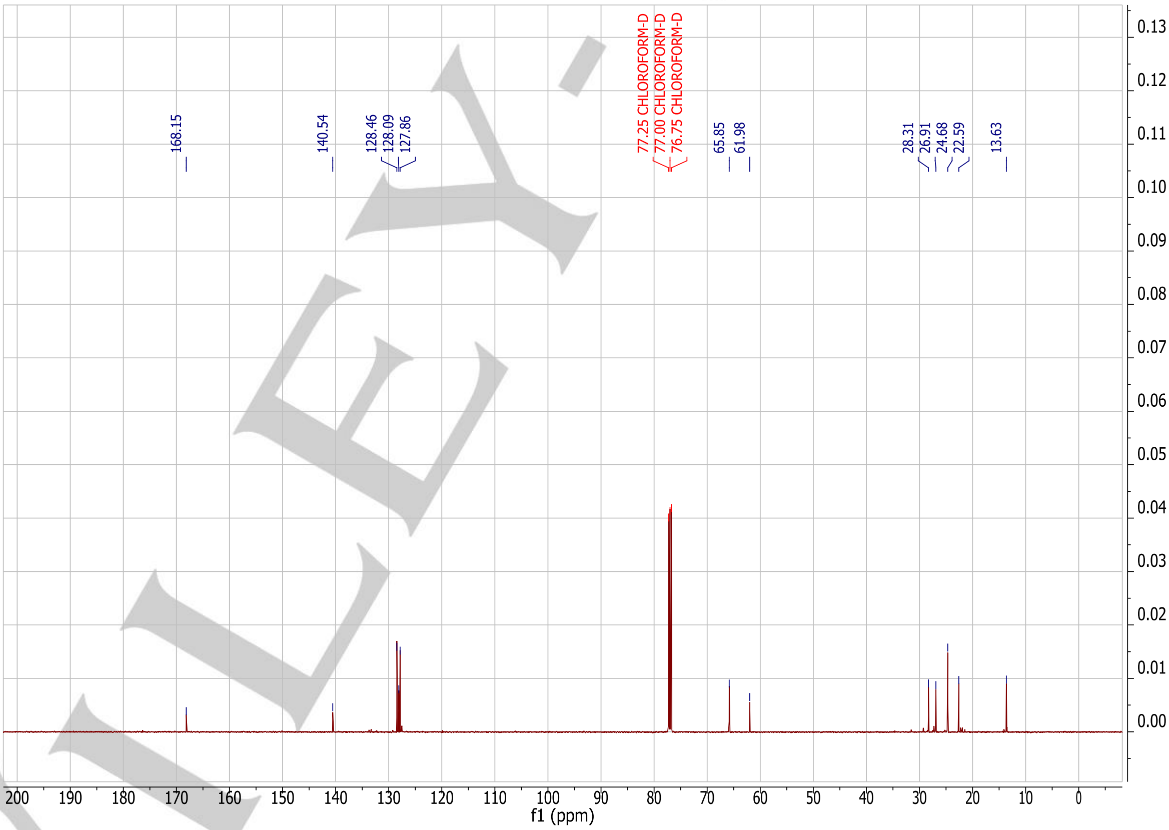

WILEY-VCH

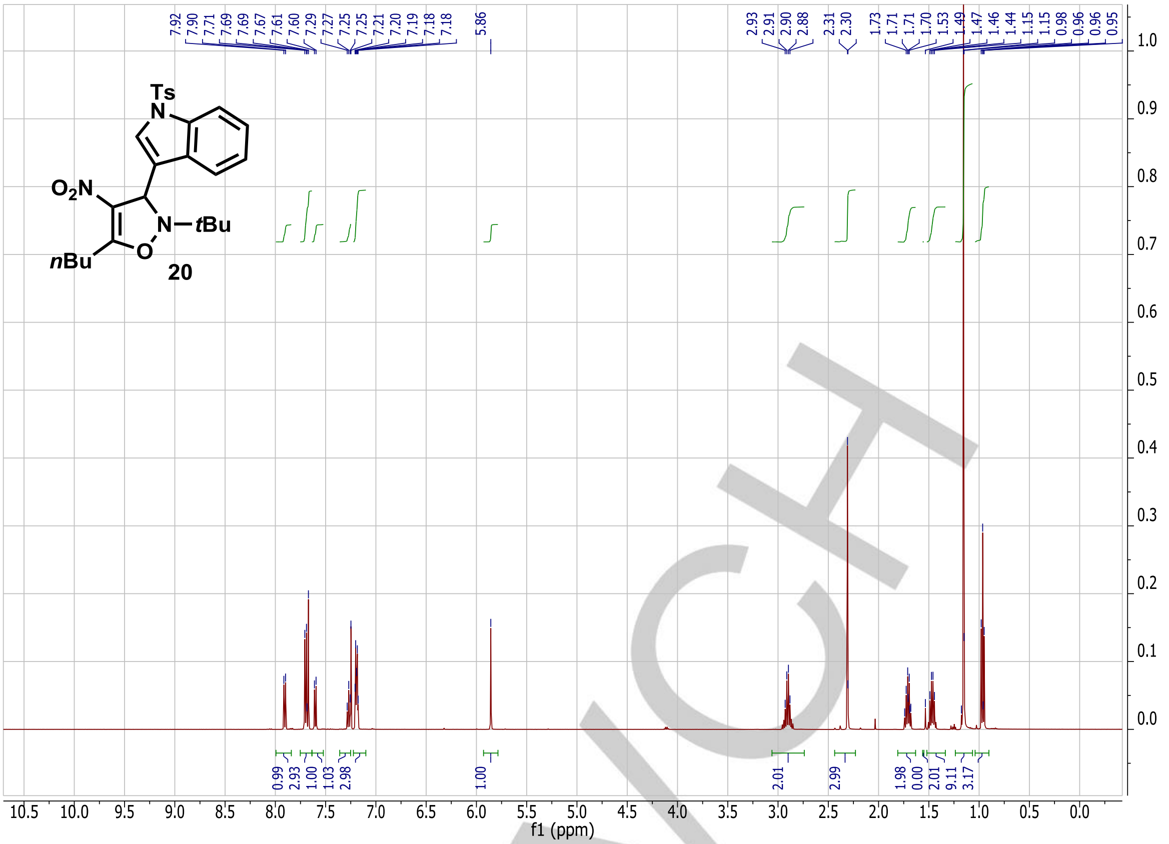
7.92 7.90 7.71 7.69 7.69 7.67 7.61 7.60 7.29 7.27 7.25 7.25 7.21 7.20 7.19 7.18 7.18
5.86
2.93 2.91 2.90 2.88 2.31 2.30 1.73 1.71 1.71 1.70 1.53 1.49 1.47 1.46 1.44 1.15 0.98 0.96 0.96 0.95
Ts
N
O2N
N—tBu
nBu
O
20
0.99 2.93 1.00 1.03 2.98
1.00
2.01 2.99 1.98 0.00 2.01 9.11 3.17
f1 (ppm)

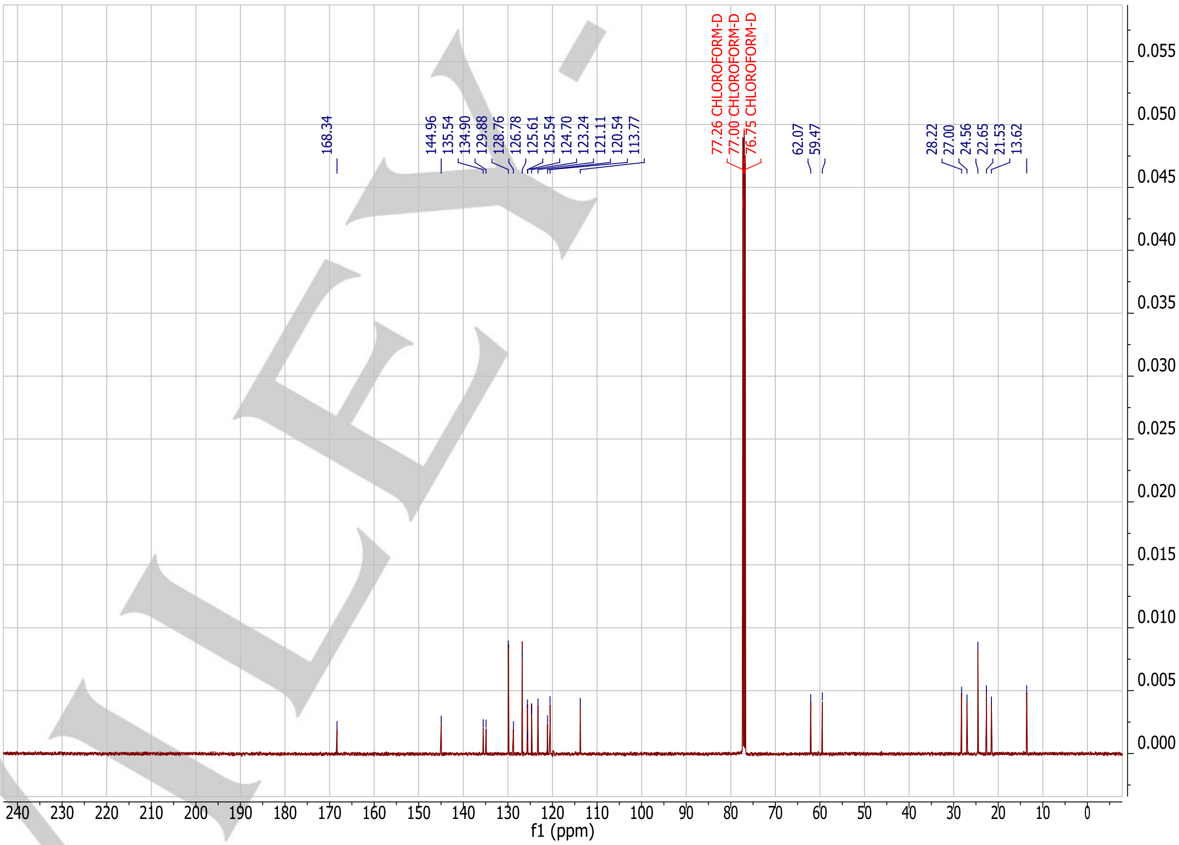
168.34
144.96 135.54 134.90 129.88 128.76 126.78 125.61 125.54 124.70 123.24 121.11 120.54 113.77
77.26 CHLOROFORM-D
77.00 CHLOROFORM-D
76.75 CHLOROFORM-D
62.07 59.47
28.22 27.00 24.56 22.65 21.53 13.62
f1 (ppm)

WILEY-VCH

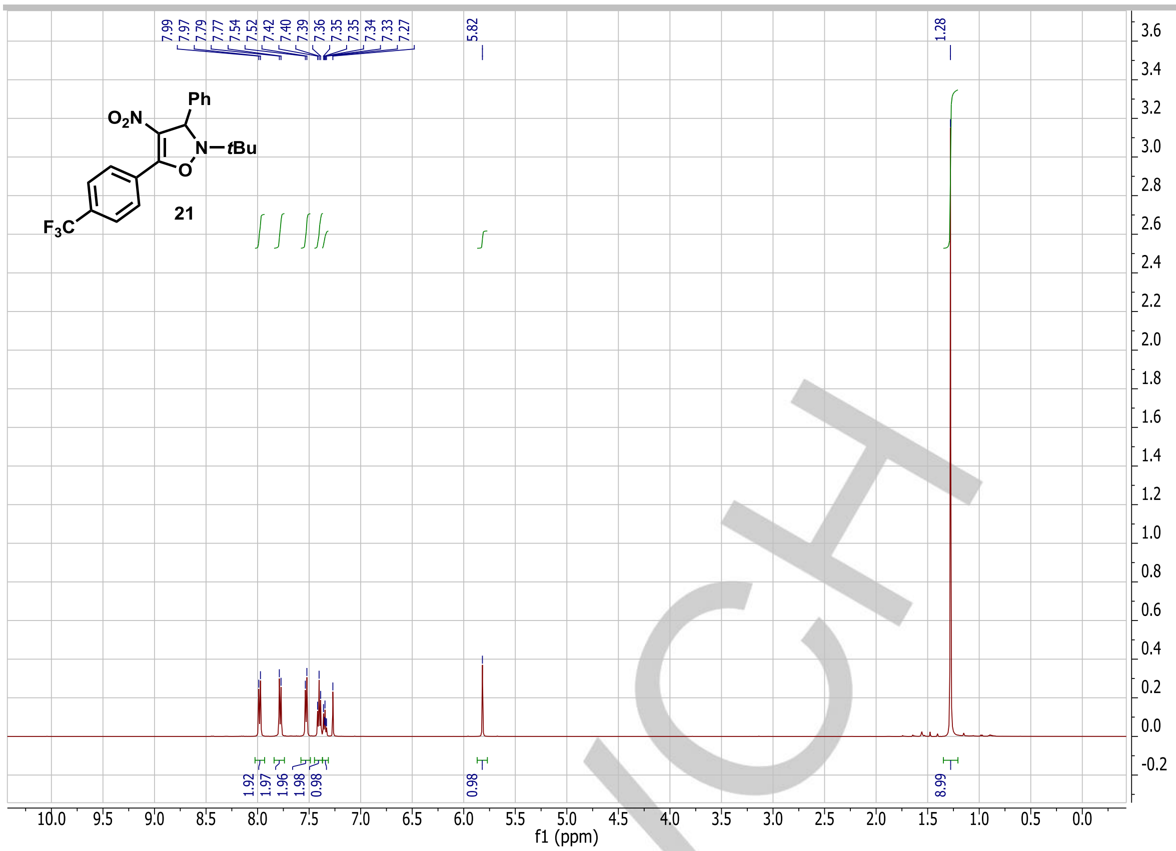
7.99
7.97
7.79
7.77
7.54
7.52
7.42
7.40
7.39
7.36
7.35
7.35
7.34
7.33
7.27
5.82
1.28
Ph
O2N
N−tBu
O
F3C
21
1.92
1.97
1.96
1.98
0.98
0.98
8.99
f1 (ppm)

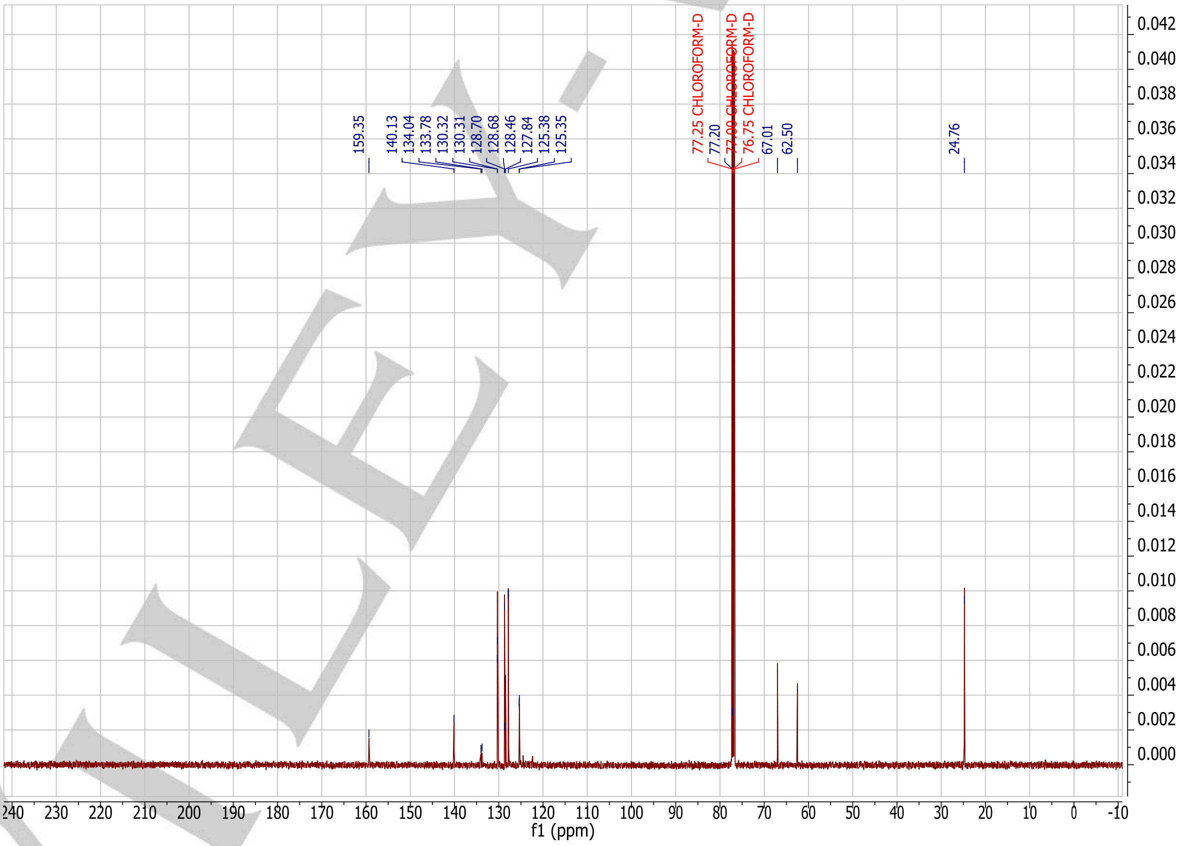
159.35
140.13
134.04
133.78
130.32
130.31
128.70
128.68
128.46
127.84
125.38
125.35
77.25 CHLOROFORM-D
77.20
77.00 CHLOROFORM-D
76.75 CHLOROFORM-D
67.01
62.50
24.76
f1 (ppm)

WILEY-VCH

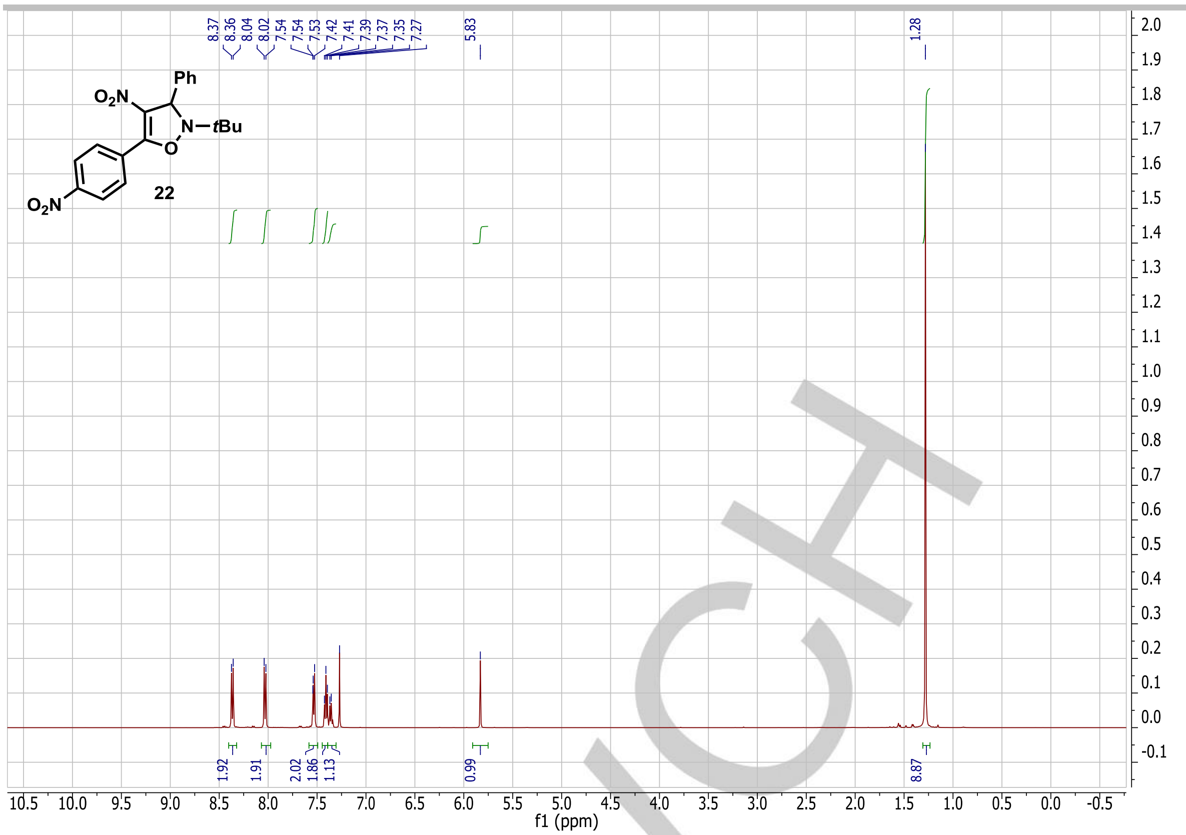
8.37
8.36
8.04
8.02
7.54
7.54
7.53
7.42
7.41
7.39
7.37
7.35
7.27
5.83
1.28
Ph
O_2N
N−*t*Bu
O
O_2N
22
1.92
1.91
2.02
1.86
1.13
0.99
8.87
f1 (ppm)

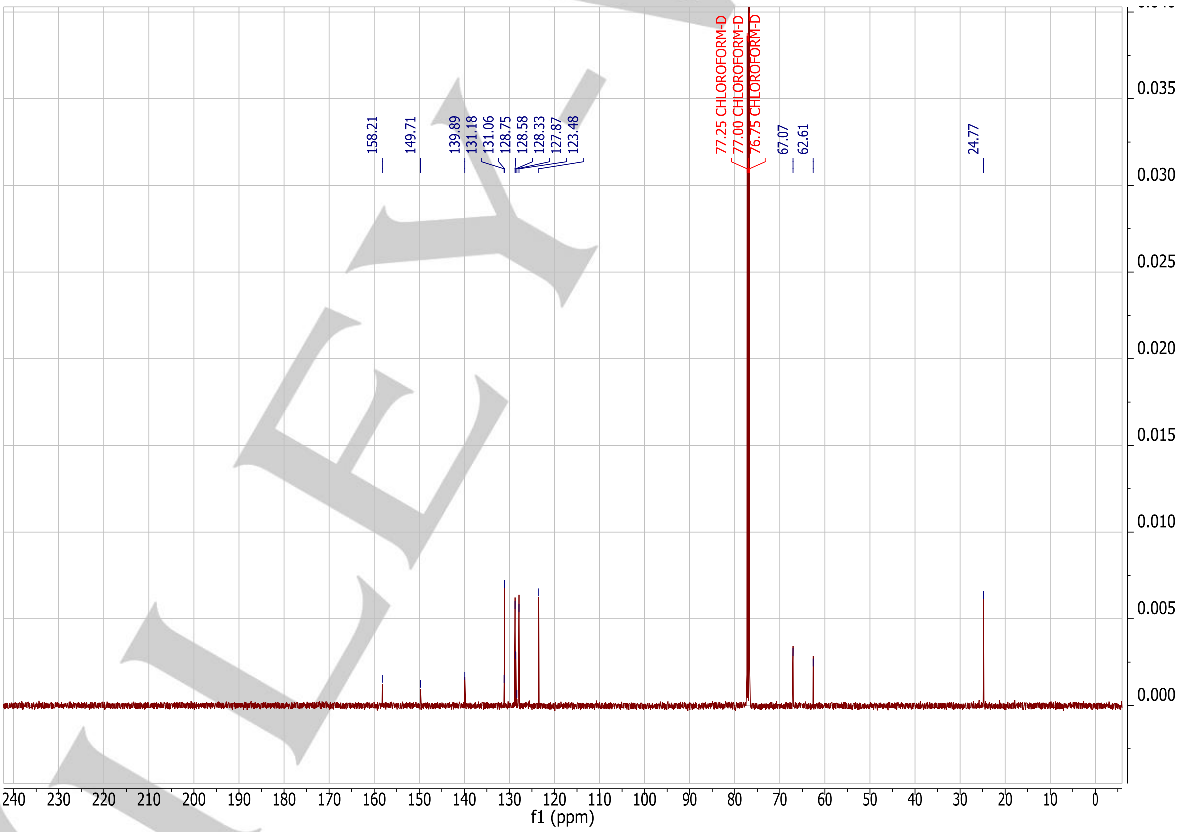
158.21
149.71
139.89
131.18
131.06
128.75
128.58
128.33
127.87
123.48
77.25 CHLOROFORM-D
77.00 CHLOROFORM-D
76.75 CHLOROFORM-D
67.07
62.61
24.77
f1 (ppm)

WILEY-VCH

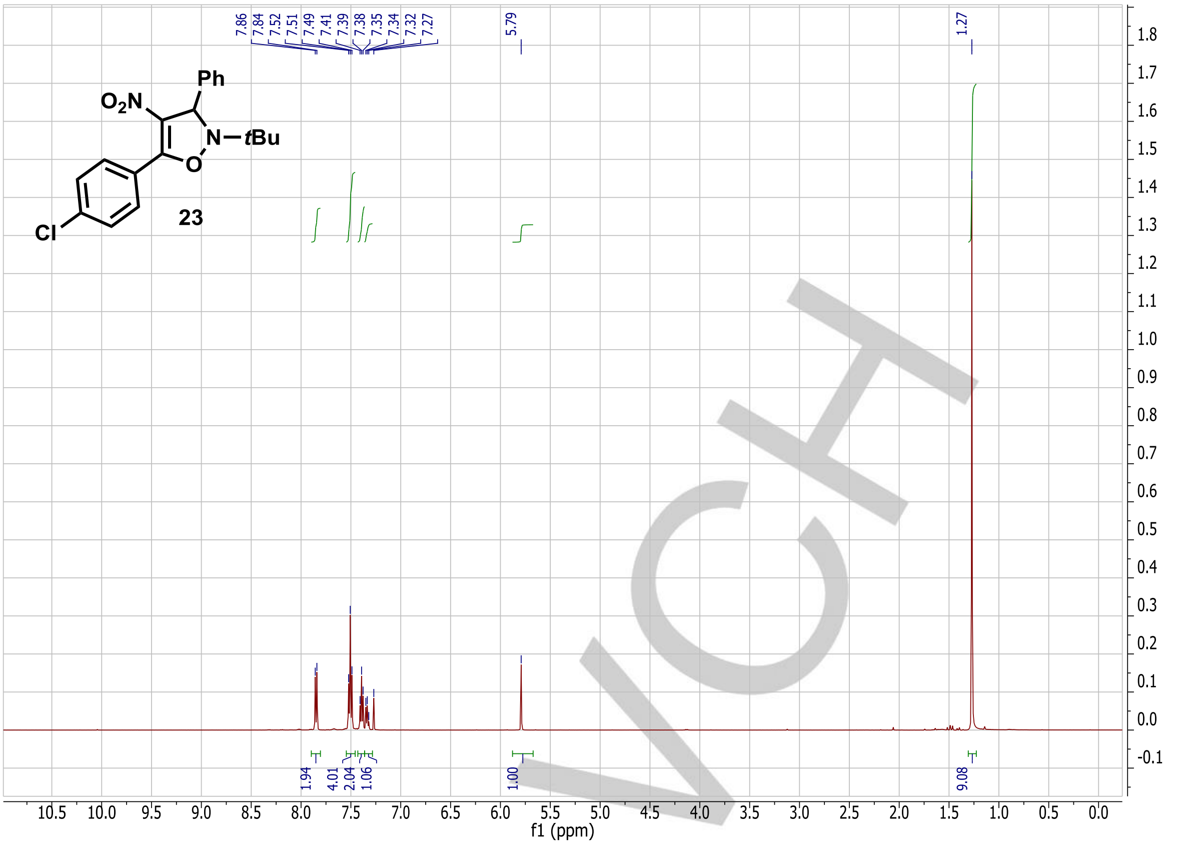
7.86
7.84
7.52
7.51
7.49
7.41
7.39
7.38
7.35
7.34
7.32
7.27
5.79
1.27
O_2N
Ph
N−tBu
O
Cl
23
1.94
4.01
2.04
1.06
1.00
9.08
f1 (ppm)

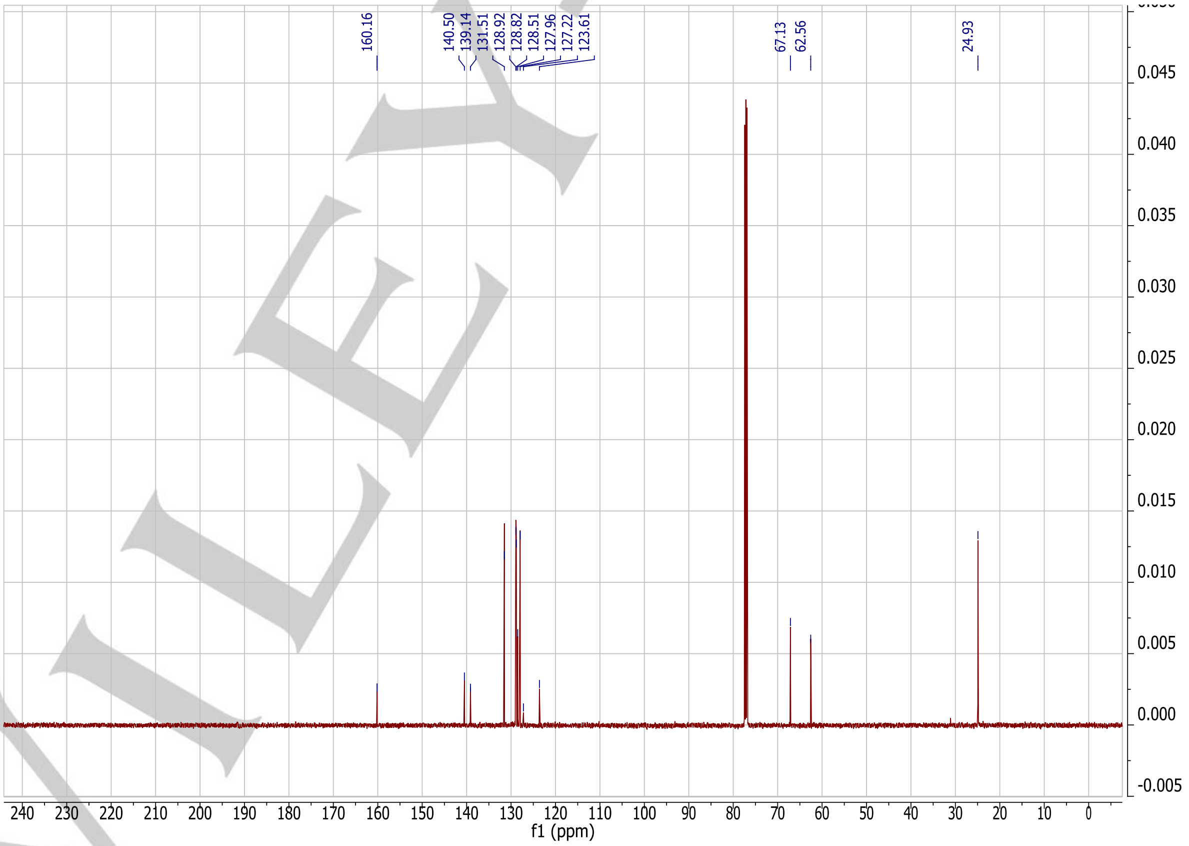
160.16
140.50
139.14
131.51
128.92
128.82
128.51
127.96
127.22
123.61
67.13
62.56
24.93
f1 (ppm)

WILEY-VCH

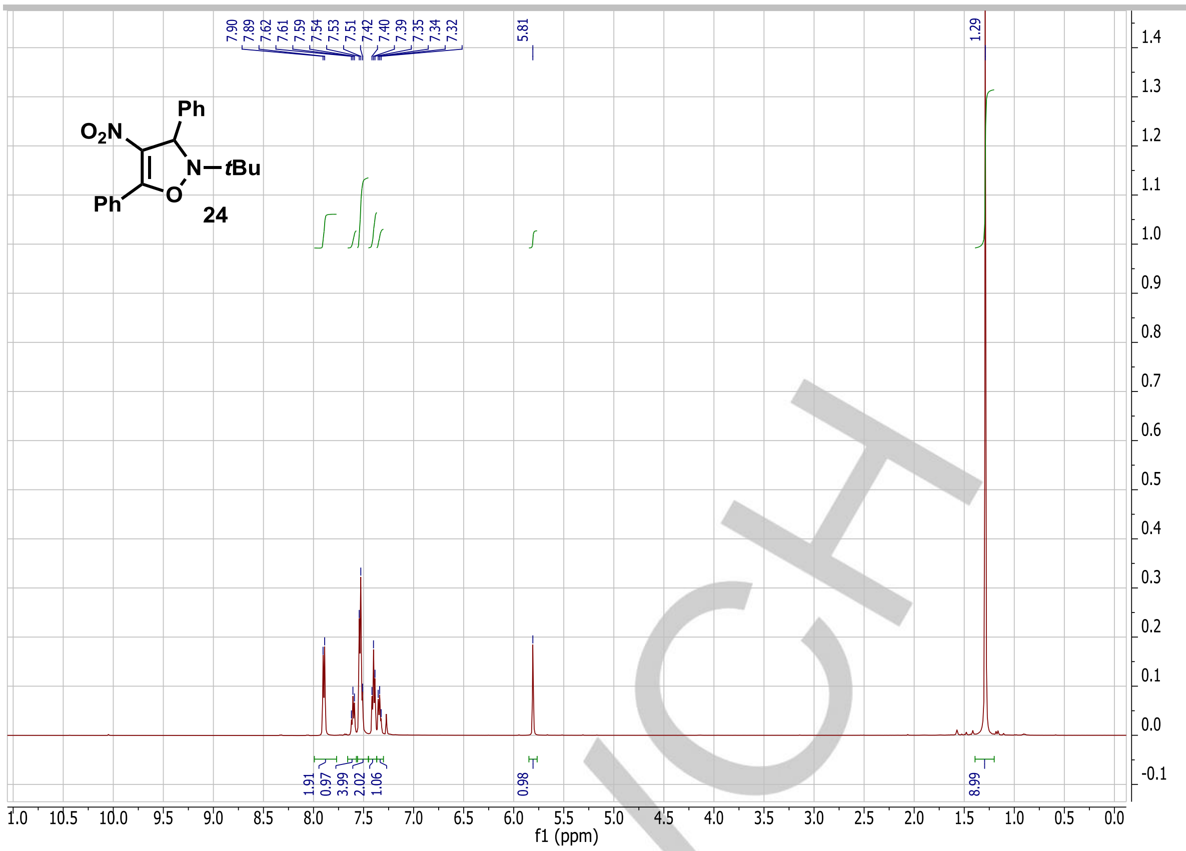

O2N
Ph
N—tBu
O
Ph
24
7.90 7.89 7.62 7.61 7.59 7.54 7.53 7.51 7.42 7.40 7.39 7.35 7.34 7.32
5.81
1.29
1.91 0.97 3.99 2.02 1.06
0.98
8.99
f1 (ppm)

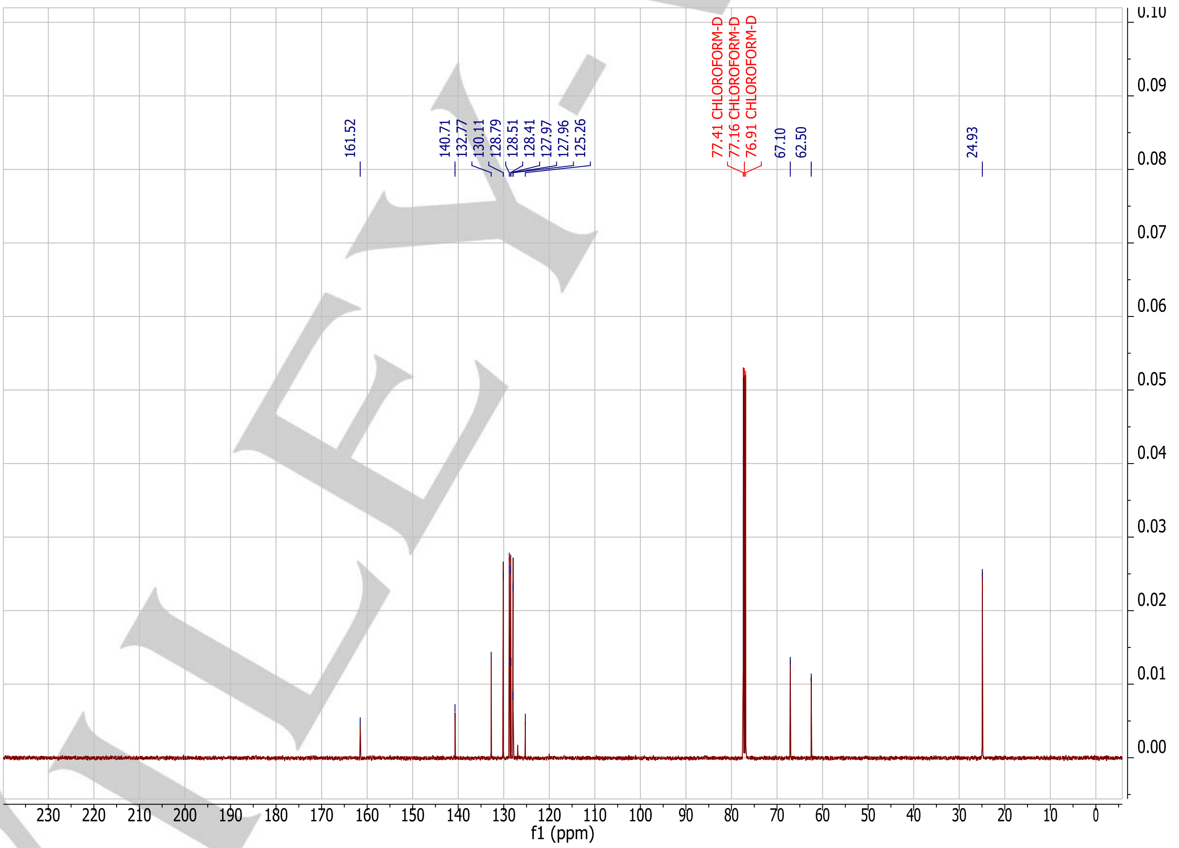

161.52
140.71 132.77 130.11 128.79 128.51 128.41 127.97 127.96 125.26
77.41 CHLOROFORM-D
77.16 CHLOROFORM-D
76.91 CHLOROFORM-D
67.10
62.50
24.93
f1 (ppm)

WILEY-VCH

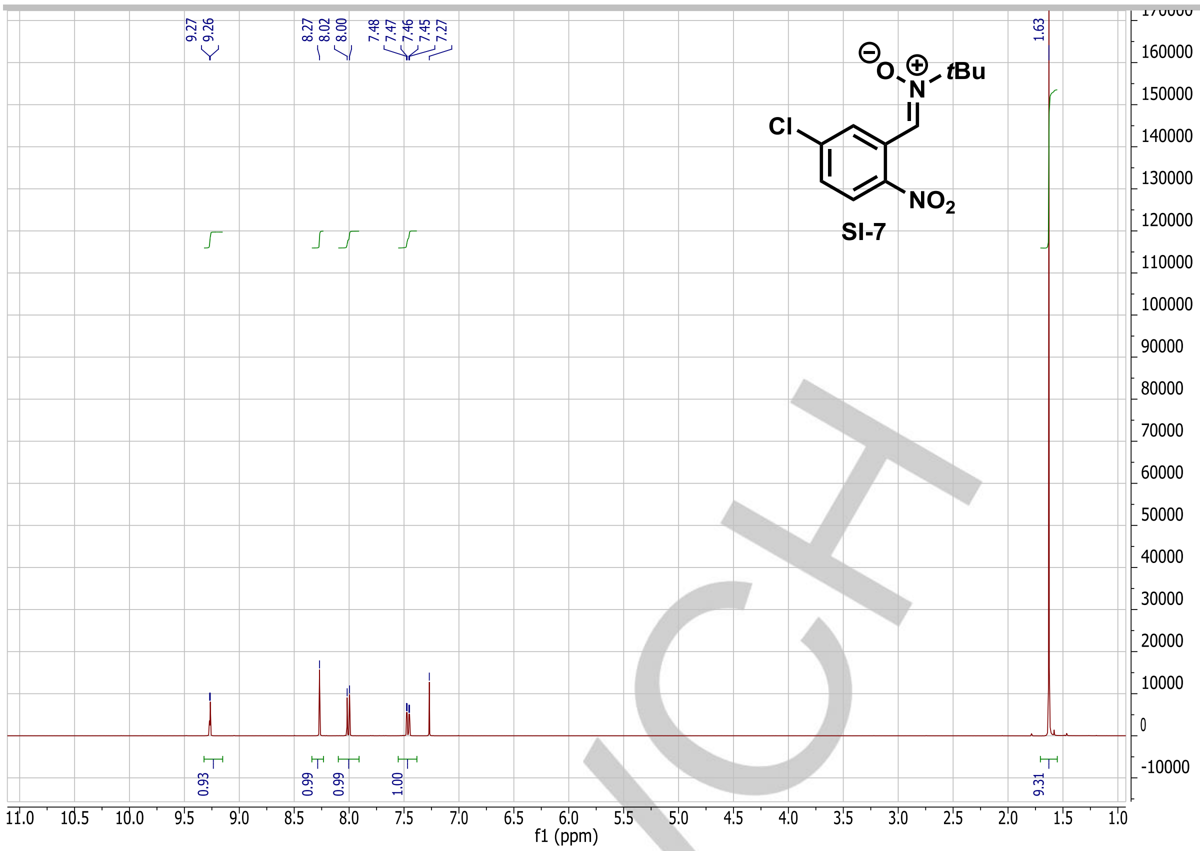
9.27
9.26
8.27
8.02
8.00
7.48
7.47
7.46
7.45
7.27
1.63
0.93
0.99
0.99
1.00
9.31
Cl
O
N
tBu
NO2
SI-7
f1 (ppm)

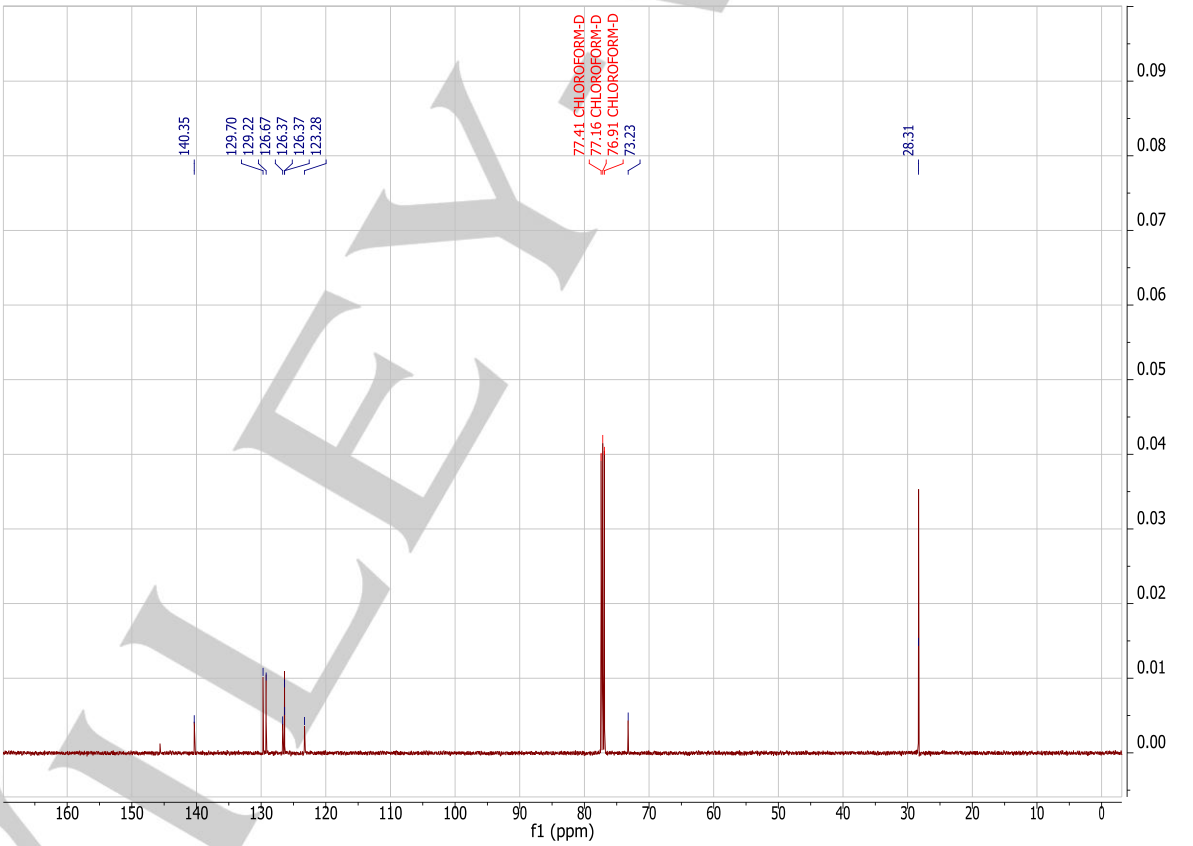
140.35
129.70
129.22
126.67
126.37
126.37
123.28
77.41 CHLOROFORM-D
77.16 CHLOROFORM-D
76.91 CHLOROFORM-D
73.23
28.31
f1 (ppm)

WILEY-VCH

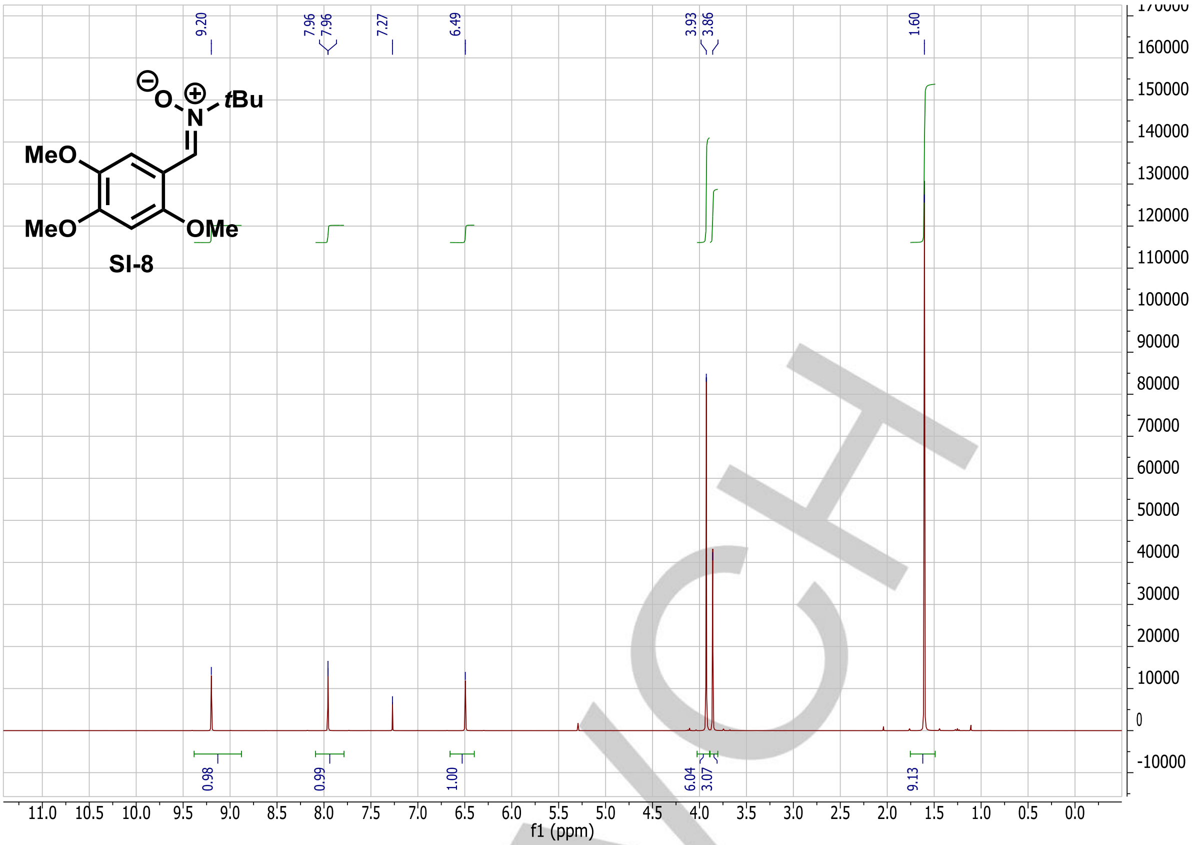
9.20
7.96
7.96
7.27
6.49
3.93
3.86
1.60
MeO
MeO
OMe
tBu
N
O
SI-8
0.98
0.99
1.00
6.04
3.07
9.13
f1 (ppm)

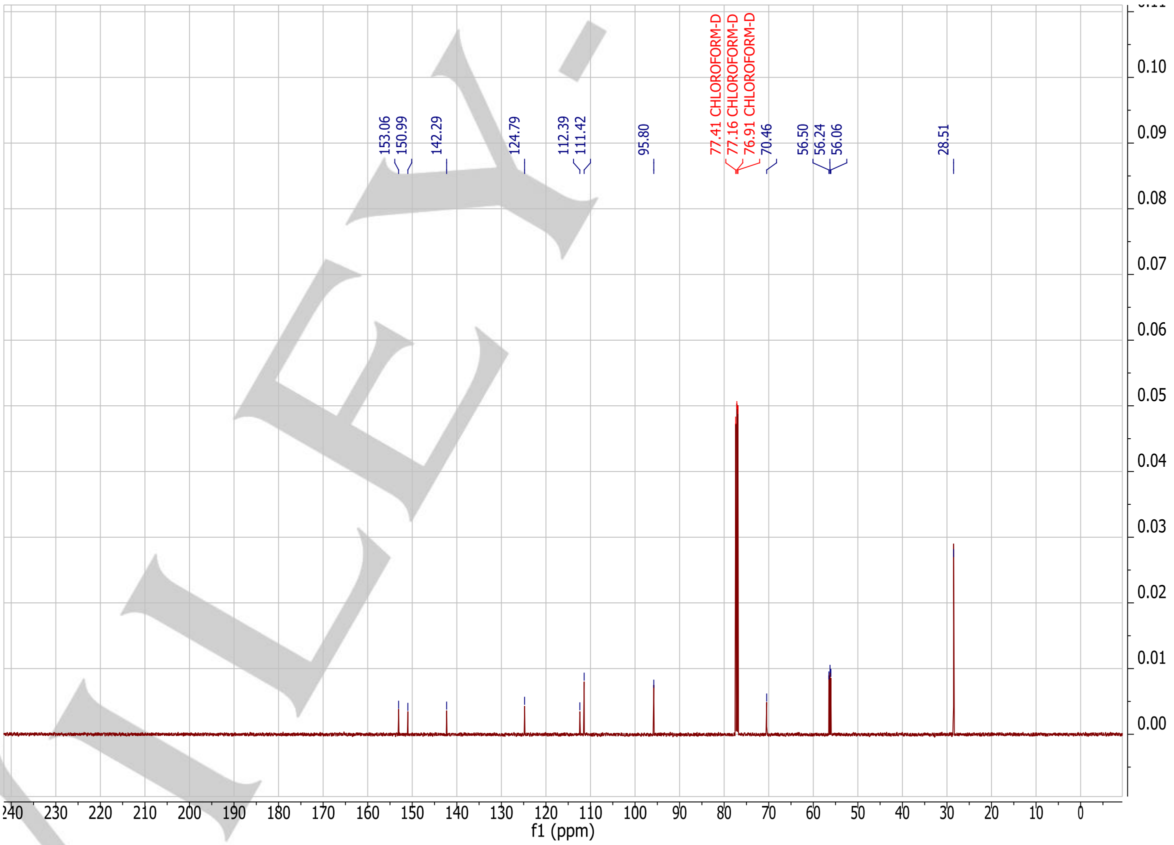
153.06
150.99
142.29
124.79
112.39
111.42
95.80
77.41 CHLOROFORM-D
77.16 CHLOROFORM-D
76.91 CHLOROFORM-D
70.46
56.50
56.24
56.06
28.51
f1 (ppm)
